# High‐Performance Prevascularized SHED‐Laden rGO@Hydrogel Achieves Optimized Diabetic Bone Defect Repair

**DOI:** 10.1002/advs.75524

**Published:** 2026-05-07

**Authors:** Can Zhang, Yiyuan Kang, Shulin Lai, Chunyi Wang, Guixin He, Kehui Jian, Suhan Yin, Xiner Tan, Xinru Zhou, Wenjing Liu, Fujian Zhao, Jia Liu, Longquan Shao

**Affiliations:** ^1^ Stomatological Hospital Southern Medical University Guangzhou China; ^2^ Guangdong Provincial Key Laboratory of Construction and Detection in Tissue Engineering Guangzhou China

**Keywords:** bone regeneration, diabetes, extracellular matrix, prevascularized scaffold, reduced graphene oxide, stem cells from the human exfoliated deciduous teeth

## Abstract

Diabetic bone defects repair is severely hindered by impaired angiogenesis and delayed osteogenesis. Conventional tissue‐engineered scaffolds often fail to achieve effective vascularization due to the compromised angiogenic capacity of host endothelial cells in the hyperglycemic microenvironment. Here, we developed a prevascularized scaffold by encapsulating stem cells from human exfoliated deciduous teeth (SHED), which shared developmental origin to craniofacial bone, within a reduced graphene oxide (rGO)‐integrated hydrogel. rGO significantly accelerated SHED‐mediated formation of vascular networks in vitro. The scaffold's therapeutic efficacy was confirmed in a clinically relevant diabetic beagle dog mandibular defect model, which showed increased vascular density and accelerated bone regeneration. Mechanistic validation revealed that rGO activates the FAK‐Src/RELA pathway to upregulate P4HA1, subsequently enhancing collagen I synthesis and driving extracellular matrix (ECM) remodeling to create a pro‐angiogenic niche. This study demonstrates that engineering the ECM with rGO is a novel strategy to accelerate prevascularization and bone repair in diabetic conditions.

## Graphical abstract

A high‐performance 3D scaffold integrating rGO and SHEDs is developed to overcome impaired healing in diabetic bone defects. By driving extracellular matrix remodeling, rGO creates a pro‐angiogenic niche that significantly accelerates in vitro vascular network formation. Upon implantation, this prevascularized construct promotes in vivo angiogenesis and osteogenesis, presenting a promising strategy for compromised diabetic regeneration.

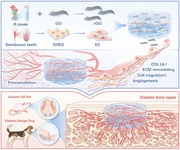

## 1 Introduction

The repair of bone defects in diabetic patients, particularly in the craniofacial region, remains a significant clinical challenge [1, 2, 3]. The diabetic microenvironment, characterized by persistent hyperglycemia, chronic inflammation, and oxidative stress [4, 5], collectively results in endothelial dysfunction and impaired angiogenesis, ultimately compromising bone healing [6, 7, 8]. This critical issue means that host‐driven neovascularization is not only slow and inefficient but also results in functionally poor and transient vessels, which ultimately undermines long‐term bone regeneration. Consequently, the implementation of robust angiogenic strategies is essential for driving functional bone tissue regeneration [9]. While conventional tissue‐engineered scaffolds aim to recruit host cells, they struggle to overcome the profound cellular dysfunction inherent to the diabetic host [10, 11, 12]. Therefore, prevascularized scaffolds, which establish a microvascular network ex vivo in 3D constructs prior to implantation, have emerged as a compelling alternative, as they can bypass the compromised host environment and facilitate rapid anastomosis with the host vasculature [13, 14, 15].

Recent studies have proved that stem cell‐based prevascularization is a leading approach to engineer these scaffolds [16, 17]. Among various cell sources, stem cells from human exfoliated deciduous teeth (SHED) are particularly advantageous for craniofacial applications due to their high proliferative capacity, low immunogenicity, and pro‐angiogenic properties [18, 19]. Critically, their shared neural crest origin with most craniofacial bones may confer a site‐specific regenerative advantage [20]. However, a significant barrier to the clinical translation of SHED‐based therapies is the protracted timeline required for vascular network formation, which typically spans several weeks of in vitro culture and limits their practical application [21, 22]. Thus, novel strategies that accelerate SHED‐mediated angiogenesis are urgently needed.

**SCHEME 1:**
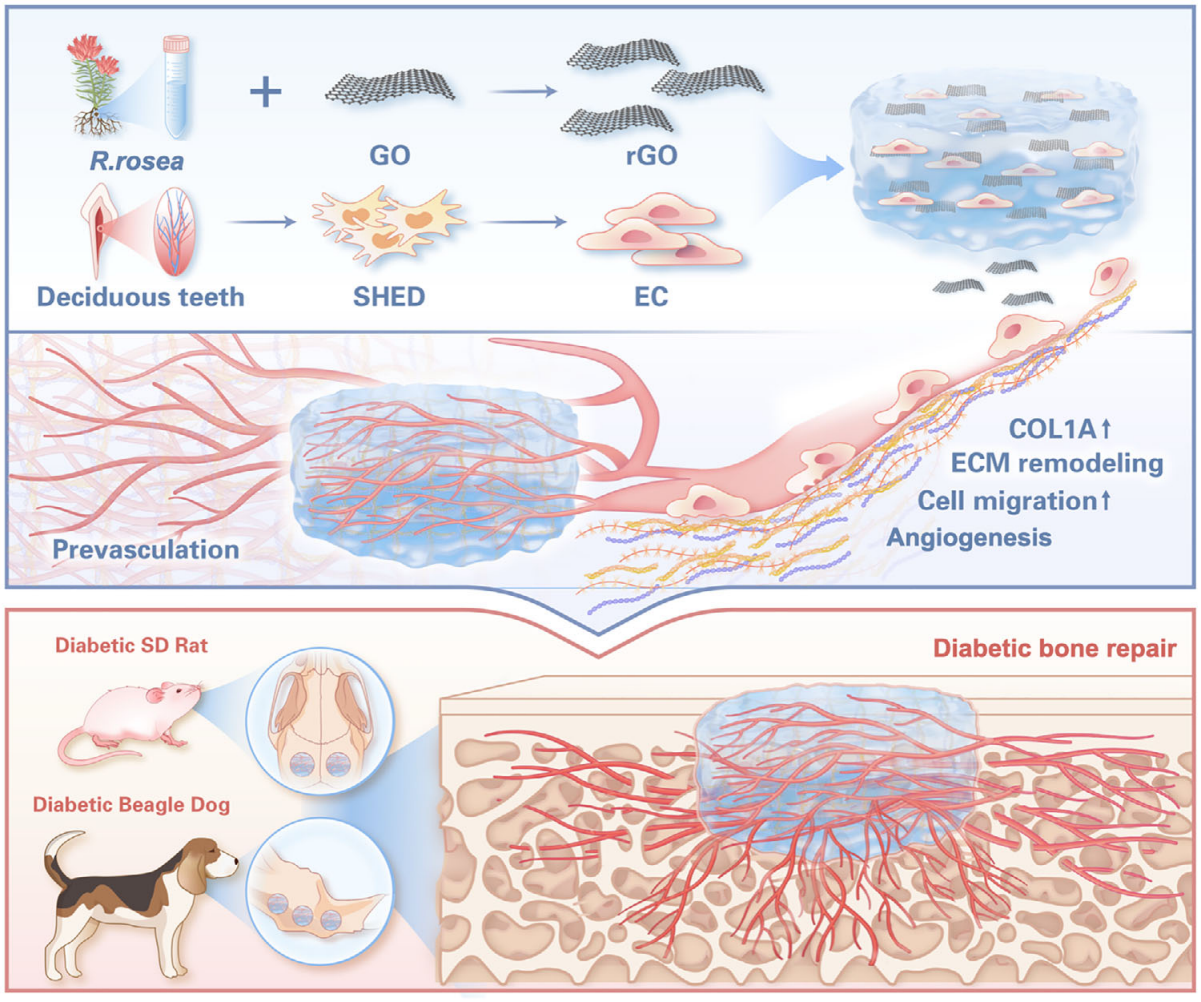
Schematic of the synthesis of the prevascularized SHED‐laden rGO@Hydrogel and its biomedical applications.

Emerging evidence suggests that nanomaterial‐based approaches hold promise for enhancing stem cell angiogenic capacity [23]. Based on the established ability of graphene‐based nanomaterials to modulate cell‐material interactions [24, 25], we demonstrated that incorporating reduced graphene oxide (rGO) into a SHED‐laden hydrogel could accelerate this prevascularization process in this study. Results obtained from the large animal models showed that these rGO‐enhanced prevascularized hydrogel scaffolds markedly accelerated bone regeneration. Distinct from prior work that predominantly focuses on growth factor delivery [26], we sought to elucidate a novel underlying mechanism, with a specific focus on how rGO influences SHED‐mediated extracellular matrix (ECM) remodeling to drive angiogenesis (Scheme 1). Taken together, our findings show that rGO acts as a bioactive cue to provide a robust and mechanistically defined strategy to overcome the profound challenge of diabetic bone repair.

## 2 Results

### 2.1 Characterization of rGO

Conventional chemical reduction of graphene oxide often involves toxic reagents, such as hydrazine hydrate, prompting a shift toward more environmentally benign, green synthesis methods [27, 28]. Among various green synthesis agents, Rhodiola rosea (*R. rosea*) was chosen for its wealth of bioactive compounds: potent antioxidants and anti‐inflammatories, which are particularly beneficial for counteracting the high oxidative stress characteristic of the diabetic milieu [29, 30]. It have been widely reported to exert cytoprotective and pro‐regenerative effects, as a medicinal plant rich in polyphenolic compounds with strong reducing and antioxidant activities [31, 32]. These properties make *R. rosea* particularly suitable not only as a green reductant for graphene oxide, but also as a biologically compatible modifier for materials intended for tissue regeneration. We therefore utilized an *R. rosea* extract for the green synthesis of rGO, with rGO reduced by hydrazine hydrate (H‐rGO) serving as a conventional control. The Fourier‐transform infrared spectroscopy revealed that the green‐synthesized rGO displayed absorption bands for hydroxyl groups (O‐H, ∼3456 cm^−1^) and carbonyl/aromatic moieties (C═O/C═C, ∼1636 cm^−1^), while the conventionally reduced H‐rGO exhibited a marked decrease in the intensity of these characteristic bands compared to GO. Furthermore, the prominent O─H and C═O/C═C signals, along with a new absorption peak at ∼679 cm^−1^ attributable to aromatic C─H bending, collectively suggested the successful anchoring of bioactive polyphenolic moieties from the extract onto the rGO surface. These surface‐bound molecules may confer additional biological benefits (Figure S1A).

**FIGURE 1:**
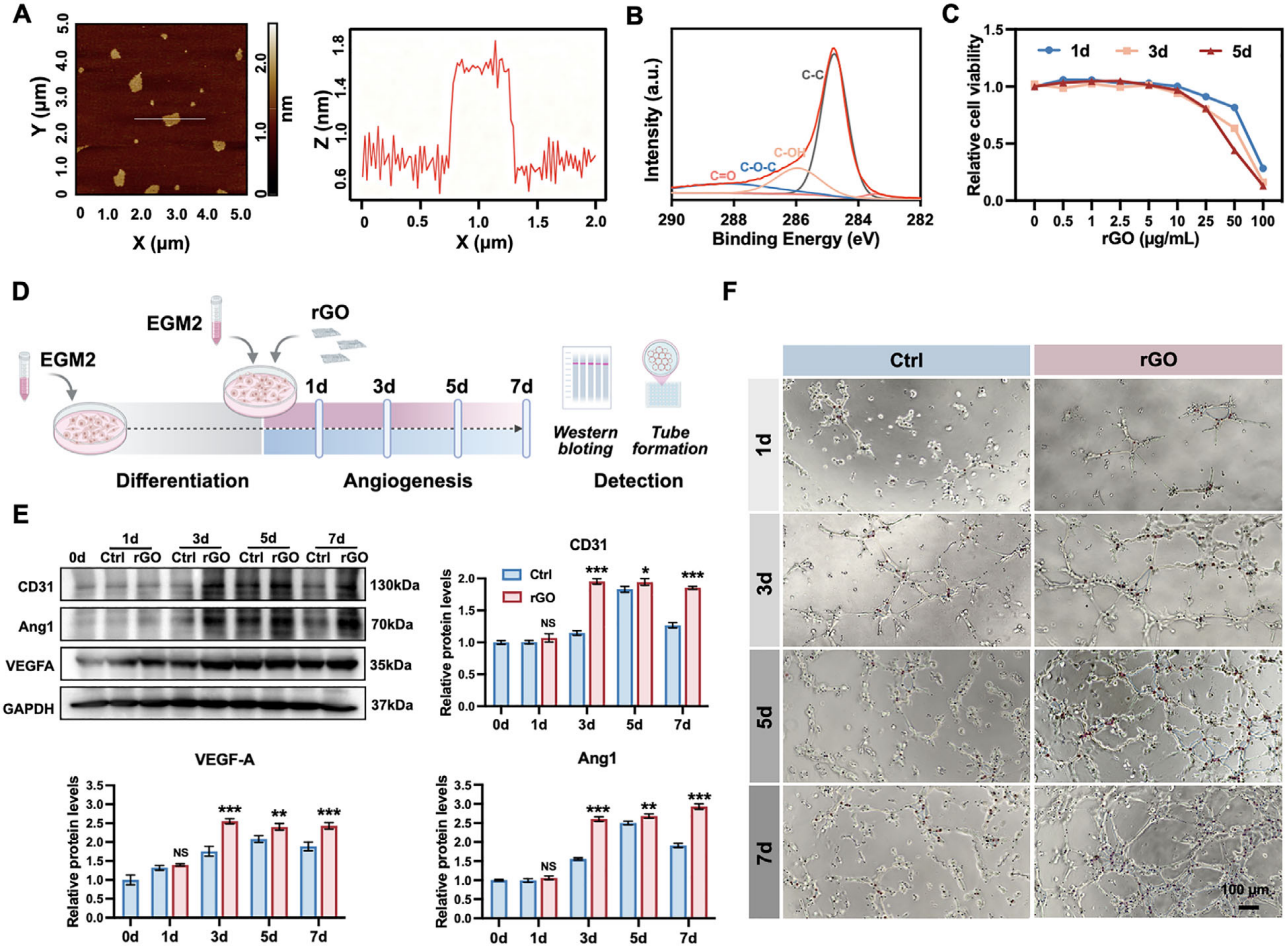
Characterization of reduced graphene oxidate (rGO) and rGO Promotes stems cell from human exfoliated deciduous teeth (SHED)‐Mediated Angiogenesis. (A) Atomic force microscopy (AFM) image showing the lateral dimensions and surface topography of an rGO sheet. (B) X‐ray photoelectron spectroscopy (XPS) spectra showing the oxygen‐containing functional groups on the rGO surface. (C) Cell counting kit‐8 (CCK‐8) assay showing the viability of SHEDs after treatment with different concentrations of rGO for 1, 3, and 5 days. (D) Schematic illustrating the experimental timeline: SHEDs were pre‐differentiated toward an endothelial lineage for 5 days, followed by treatment with 5 µg/mL rGO to assess angiogenic induction. Schematic illustrations created with BioRender.com. SHEDs were endothelial differentiated for 5 days, cells were then harvested and treated with 0 and 5 µg/mL rGO for different time periods. (E) Western blot analysis of angiogenesis‐related proteins (CD31, Ang1, VEGF‐A) in SHEDs after treatment with rGO for 0, 1, 3, 5, and 7 days. (F) Representative images of tube formation assays of SHEDs treated with rGO for 1, 3, 5, and 7 days. Scale bar, 100 µm. Data in (E) are presented as mean ± SD. (*n* = 3 per group). Statistical significance was determined using a two‐way analysis of variance (ANOVA) followed by Sidak's multiple comparisons test. ^*^ *p* < 0.05, ^**^ *p* < 0.01 and ^***^ *p* < 0.001.

The physicochemical properties of rGO are critical to its biological effects [33]. Transmission electron microscopy (TEM) confirmed the typical sheet‐like morphology of the synthesized rGO (Figure S1B). Atomic force microscopy (AFM) revealed lateral dimensions of approximately 400 nm and a heterogeneous surface topography with a maximum thickness of ∼25 nm (Figure 1A). To mitigate the known tendency of graphene‐based nanomaterials to aggregate in physiological media, all rGO dispersions were thoroughly ultrasonicated prior to use. Raman spectroscopy yielded a D‐band to G‐band intensity ratio (ID/IG) of 0.85, indicating a high degree of graphitization with minimal structural defects. The ID /IG ratio for H‐rGO was 1.30, suggesting that a more disordered structure was induced by the harsh chemical process (Figure S1C). X‐ray photoelectron spectroscopy (XPS) analysis showed that the oxygen atomic percentage was 10.66% for rGO and 11.19% for H‐rGO, confirming successful reduction (Figure 1B; Figure S1D) [34]. Furthermore, x‐ray diffraction (XRD) showed a characteristic broad diffraction peak at 2*θ* = 25.94° corresponding to the (002) plane of graphene, while H‐rGO exhibited a corresponding peak at a slightly lower angle of 2*θ* = 25.64°, indicative of a similar interlayer spacing. (Figure S1E).

The biological performance of rGO was subsequently evaluated. Cell Counting Kit‐8 (CCK‐8) assays demonstrated excellent cytocompatibility for the green‐synthesized rGO, with concentrations up to 10 µg/mL showing no adverse effect on SHED viability. In contrast, H‐rGO exhibited significant cytotoxicity, with cell viability beginning to decline at a concentration as low as 1 µg/mL and decreasing further in a dose‐dependent manner (Figure 1C; Figure S1F). In addition, rGO synthesized via *R. rosea*‐mediated green reduction effectively attenuated H_2_O_2_‐induced extracellular reactive oxygen species (ROS) accumulation in SHEDs, indicating favorable antioxidant activity. Conversely, H‐rGO not only failed to reduce ROS levels but appeared to slightly elevate them (Figure S1G). Collectively, these characterizations confirm the successful green synthesis of rGO with well‐defined structural features, low cytotoxicity, and good antioxidant properties. Thus, the green method with *R. rosea* not only achieved a reduction efficiency comparable to chemical standards, but also simultaneously functionalized the rGO surface with intrinsic ROS‐scavenging activity. This engineering creates an rGO variant capable of remediating local pathology, a design specifically targeted at the challenging diabetic microenvironment.

### 2.2 rGO Promotes SHED‐Mediated Angiogenesis

As the seed cell is a key component of a tissue engineering scaffold, we strategically selected SHED for its homologous origin to craniofacial tissues, multi‐lineage potential, and high translational promise [18, 19]. We obtained SHEDs and then confirmed the mesenchymal stem cell identity by flow cytometry, which showed positive expression for CD105, CD73, and CD90, and negative expression for CD34, CD45, and HLA‐DR (Figure S2). Based on a time‐course analysis of endothelial marker expression, a 5‐day induction period in EGM‐2 medium was determined to be optimal for priming SHEDs toward an endothelial lineage (Figure S3). Following this 5‐day pre‐differentiation, the cells were treated with the *R. rosea*‐synthesized rGO to assess its effect on angiogenesis (Figure 1D). A dose‐response study using a tube formation assay identified 5 µg/mL as the optimal concentration, as it induced the most significant increase in tube nodes and master segment length (Figure S4A).

At this optimal concentration (5 µg/mL), rGO treatment resulted in a time‐dependent enhancement of angiogenesis. This was evidenced by significantly improved tube formation capacity starting from day 3 (Figure 1F; Figure S4B). Consistent with these morphological changes, western blot and quantitative real‐time PCR (qRT‐PCR) analyses revealed a progressive upregulation of key angiogenic markers, including CD31, Angiopoietin‐1 (Ang1), and VEGF‐A, at both the protein and mRNA levels (Figure 1E; Figure S4C). Together, these findings demonstrate that rGO at 5 µg/mL effectively accelerates SHED‐mediated angiogenesis in vitro.

### 2.3 The Prevascularized SHED‐Laden rGO@Hydrogel Effectively Promotes Angiogenesis and Osteogenesis in Diabetic Beagle Bone Defects

**FIGURE 2:**
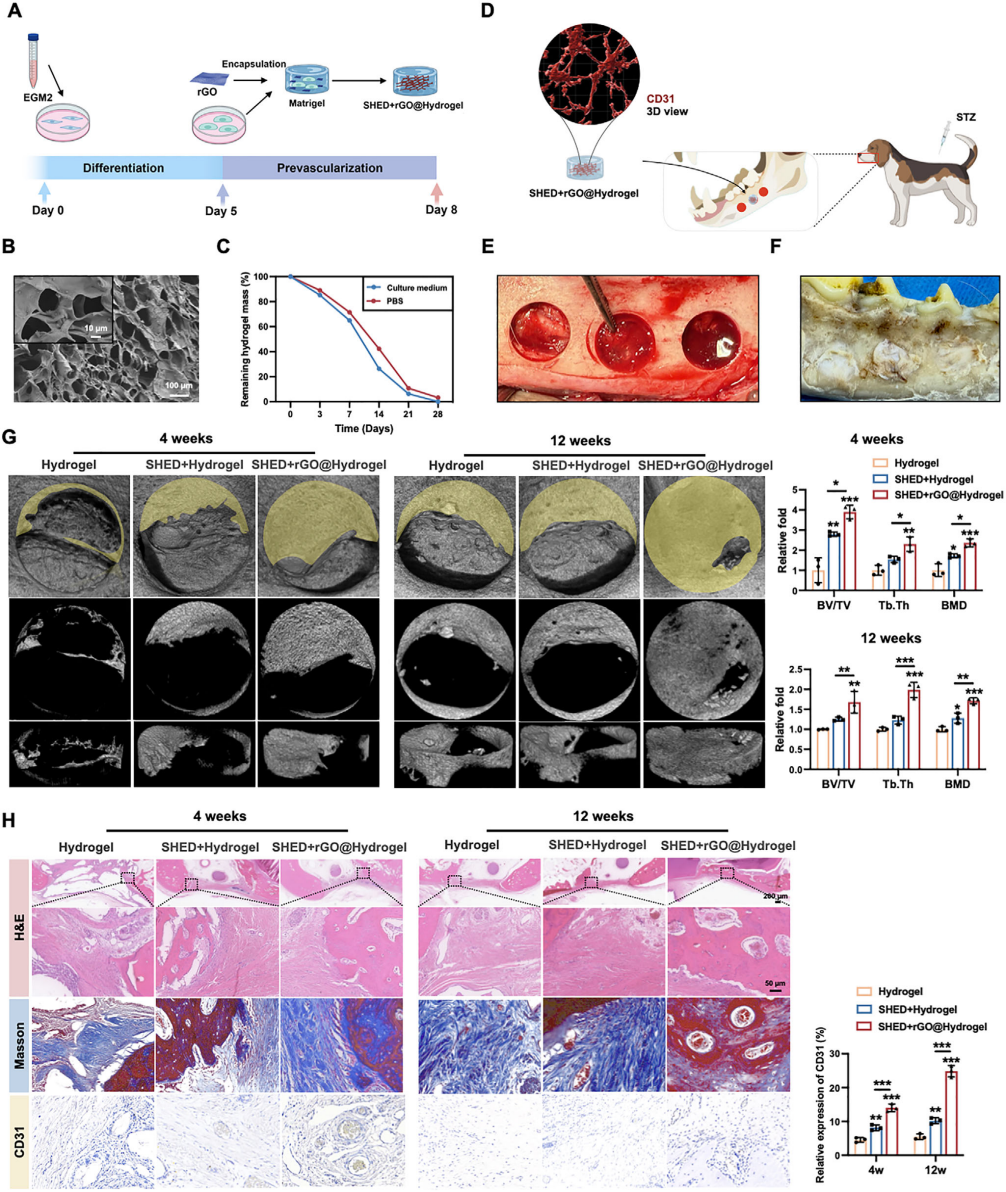
The prevascularized SHED‐laden rGO@Hydrogel effectively promotes angiogenesis and osteogenesis in diabetic beagle bone defects. (A) Schematic illustration the fabrication of the SHED‐laden rGO@Hydrogel, its in vitro prevascularization, and subsequent implantation into a diabetic beagle dog mandibular defect model. (B) Representative scanning electron microscopy (SEM) image of the rGO@Hydrogel, showing its porous microstructure. Scale bar, 100 µm. (C) In vitro degradation profile of the rGO@Hydrogel in PBS and culture medium over 28 days. (D) Representative 3D reconstruction of CD31 immunofluorescence images showing vascular‐like network formation by SHEDs cultured in hydrogels with rGO. And establishment of a critical‐sized mandibular bone defect in beagle dogs followed by implantation of the prevascularized SHED+rGO@Hydrogel scaffold. (E) Surgical procedure showing the creation of a critical‐sized mandibular defect in a beagle dog and implantation of the scaffold. (F) Gross view of harvested mandible specimens harvested at 4 and 12 weeks for subsequent tests. (G) Micro‐CT reconstructions and quantitative analysis of bone volume/tissue volume (BV/TV), trabecular thickness (Tb. Th) and bone mineral density (BMD) at 4 and 12 weeks. following implantation of the prevascularized SHED+rGO@Hydrogel scaffold. (H) Representative images of H&E staining, Masson staining, and CD31 immunohistochemistry of the defect area at 4 and 12 weeks. Scale bars, 200 µm (overview) and 50 µm (magnified inset). Data in (G) and (H) are presented as mean ± SD (*n* = 3 per group). Statistical significance was determined using one‐way ANOVA followed by Tukey's *post hoc* test. ^*^ *p*<0.05, ^**^ *p*<0.01 and ^***^ *p*<0.001.

To engineer a prevascularized scaffold, we first developed a hydrogel integrated with reduced graphene oxide (rGO) to serve as a bioactive carrier for SHEDs (Figure 2A). The scaffold's microstructure and degradation kinetics are critical determinants of cell behavior and subsequent tissue integration. Scanning electron microscopy (SEM) revealed that the rGO@Hydrogel possessed a highly porous and interconnected network with a mean pore diameter of 72.73 µm (Figure 2B), which falls within the 50–150 µm range reported to be ideal for nutrient diffusion and cell migration [35, 36]. In vitro degradation assays demonstrated a progressive mass loss, with approximately 50% of the hydrogel mass remaining at day 14 and complete degradation by day 28 (Figure 2C). This degradation profile is well‐suited to provide initial structural support during the critical phase of vascularization, followed by gradual resorption to allow for host tissue infiltration and remodeling [37].

We next evaluated the capacity of rGO to accelerate the formation of vascular‐like networks by encapsulated SHEDs, a key step in creating the prevascularized construct. High cell viability was confirmed across all hydrogel groups via live/dead staining, with the rGO@Hydrogel group showing minimal cell death (Figure S5). Crucially, 3D reconstruction of CD31 immunofluorescence staining after 3 days of culture revealed that SHEDs within the rGO@Hydrogel formed a significantly more extensive and organized vascular‐like network. This was evidenced by a higher density of interconnected tubules and an increased number of branch points compared to the control hydrogel without rGO, indicating that rGO effectively accelerates in vitro prevascularization (Figure 2D).

To assess the translational efficacy of this strategy, we utilized a clinically relevant, large‐animal diabetic model. The beagle dogs successfully established a stable diabetic phenotype, characterized by persistent hyperglycemia, polydipsia (increased water intake), and significant body weight loss (Figure S6). In these animals, the prevascularized SHED‐laden rGO@Hydrogel was implanted into a critical‐sized mandibular defect (Figure 2E,F). At 4‐ and 12‐ weeks post‐implantation, micro‐CT analysis showed substantially greater new bone formation in the SHED+rGO@Hydrogel group. This group exhibited significantly higher bone volume/tissue volume (BV/TV), and bone mineral density (BMD) compared to the SHED+Hydrogel control group (Figure 2G). Histological analysis corroborated these quantitative findings. Hematoxylin and eosin (H&E) staining revealed thicker, more mature bone trabeculae, while Masson's trichrome staining indicated denser and more organized collagen deposition in the SHED+rGO@Hydrogel group. Consistent with our prevascularization strategy, CD31 immunohistochemistry confirmed a significantly higher microvessel density in defects treated with the SHED+rGO@Hydrogel (Figure 2H). Collectively, these results demonstrate that the rGO‐enhanced prevascularized scaffold effectively promotes coupled angiogenesis and osteogenesis in a challenging diabetic large‐animal model. However, the underlying molecular mechanisms by which rGO accelerates SHED‐mediated vascularization require elucidation.

### 2.4 rGO Enhances SHED Angiogenesis by Promoting Cell Migration Through ECM Remodeling

**FIGURE 3:**
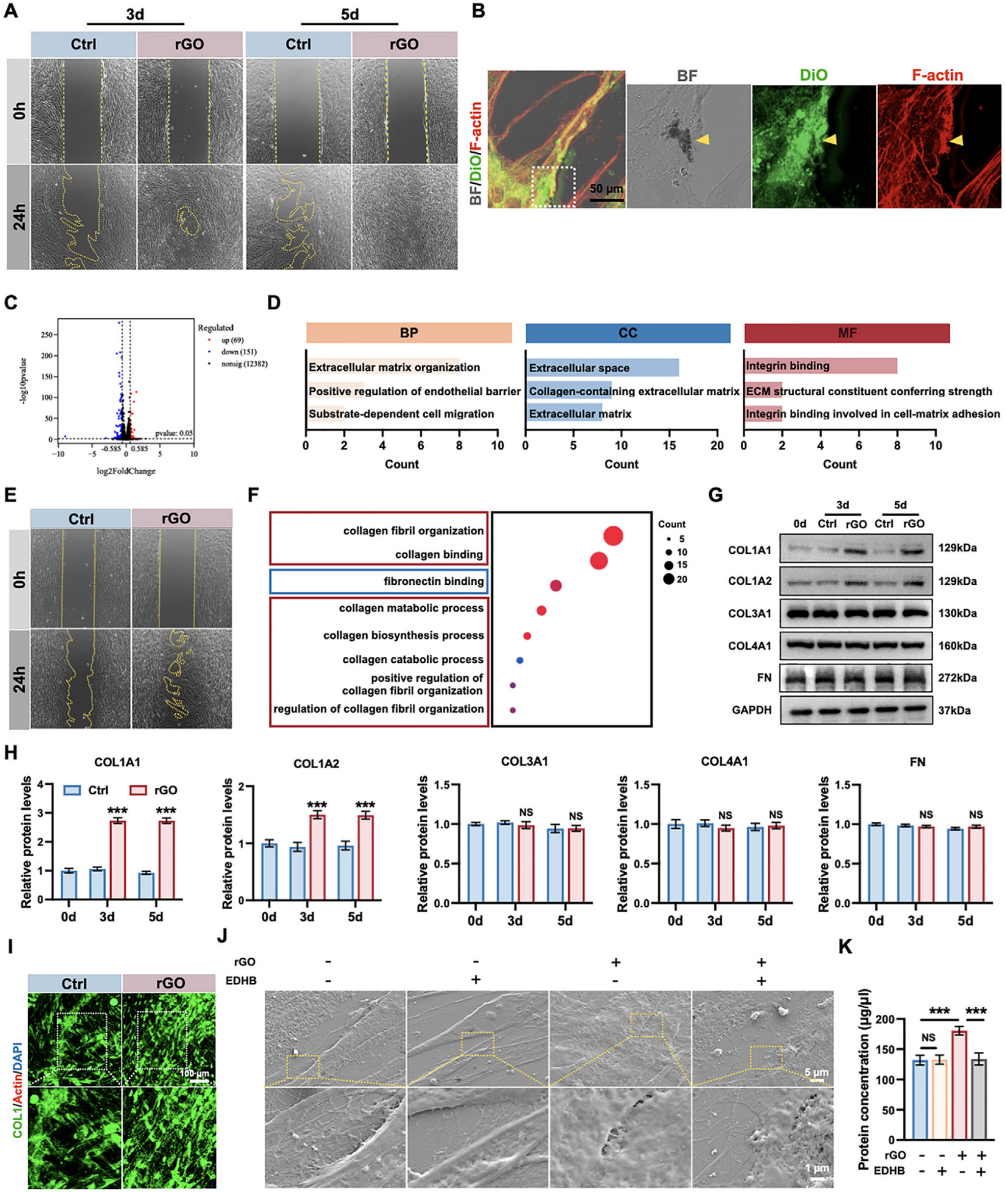
rGO Enhances SHED angiogenesis by promoting cell migration through Collagen I–driven ECM remodeling. (A) Representative images and quantification of a wound healing assay in SHEDs after treatment with rGO for 3 and 5 days. The wound area (%) was measured using ImageJ. (B) Confocal microscopy images showing colocalization (yellow arrows) of rGO (bright field, BF) with the cell membrane (Dio staining, green) and F‐actin (phalloidin staining, red). Scale bar, 50 µm. (C) Volcano plot of differentially expressed genes (DEGs) expression analysis of SHEDs treated with rGO for 2 days. (D) Gene Ontology analysis enrichment of DEGs, showing top terms for biological process, cellular component, and molecular function. (E) Wound healing assay in SHED cultured on decellularized ECM derived from either control or rGO‐treated cells. (F) Gene Ontology analysis of ECM‐related DEGs, with collagen‐related genes highlighted in red and fibronectin in blue. (G,H) Western blot analysis and quantification of collagen I (COL1A1 and COL1A2), III, IV, and fibronectin in SHEDs treated with rGO for 3 and 5 days. (I) Immunofluorescence staining of type I collagen in the decellularized ECM from control and rGO‐treated SHEDs. Scale bar, 100 µm. (J,K) SEM imaging and BCA quantification of total protein in decellularized ECM from SHEDs treated with rGO with or without the collagen I inhibitor EDHB. Scale bar, 5 µm (overview) and 1 µm (magnified inset). Data in (K) are presented as mean ± SD. (*n* = 3 per group). Statistical significance was determined using a two‐way analysis of variance (ANOVA) followed by Sidak's multiple comparisons test for panel H and by one‐way ANOVA followed by Tukey's *post hoc* test for panels K. NS: no significance, ***p < 0.001.

Angiogenesis is a multi‐step process reliant on endothelial cell proliferation and migration. Our 5‐ethynyl‐2’‐deoxyuridine (EdU) incorporation assays revealed no significant difference in the proportion of proliferating cells between the rGO and control groups (Figure S7A,B). In contrast, a wound healing assay demonstrated a significant acceleration of wound closure in the rGO‐treated group at both day 3 and day 5. These results indicate that rGO promotes angiogenic effect primarily through enhancing SHED migratory capacity but not by enhancing cell proliferation (Figure 3A; Figure S7C). Given that cell migration is fundamentally driven by cytoskeletal dynamics [38], and 2D nanomaterials, such as rGO, exhibit a strong affinity for the cell membrane and its associated structures. Thus, we investigated the physical interaction between rGO and SHEDs using scanning electron microscopy (SEM) and immunofluorescence. SEM imaging showed that rGO nanosheets initially localized to the cell membrane (Figure S8A), acting as an external mechanical stimulus that could cause membrane curvature, inducing the remodeling of the actin cytoskeleton [39]. In our study, dual fluorescence staining of the membrane and filamentous actin (F‐actin) revealed that rGO first contacts the plasma membrane, triggering a rapid and localized reorganization of the underlying cytoskeleton. Specifically, we observed the condensation of F‐actin at the material‐cell interface (Figure 3B). After rGO treatment for 3 h, the fluorescence images showed that the internalization of rGO was coupled with the formation of prominent, thick actin stress fibers along the cell membrane, indicating a sustained and evolving cytoskeletal response to the material (Figure S8B). These results suggest that rGO modulates cellular responses by initially acting as a mechanobiological cue at the cell surface to regulate actin dynamics. While these methods primarily provide indirect or static insights, advanced techniques that can capture more direct and dynamic changes in the cell membrane in response to mechanical stimuli would be invaluable in future investigations [40].

To elucidate the underlying molecular mechanism, we performed RNA sequencing (RNA‐seq) on SHEDs treated with rGO. Gene Ontology enrichment analysis of differentially expressed genes (DEGs) revealed a strong enrichment of terms related to “extracellular matrix (ECM) organization”, “cell migration”, and “collagen‐containing ECM” (Figure 3C,D). To functionally validate this transcriptomic signature, we performed a decellularized matrix (dECM) experiment. ECM produced by rGO‐treated SHEDs significantly enhanced the migration of SHEDs compared to ECM from control cells, confirming that rGO modulates cell migration by remodeling the ECM (Figure 3E; Figure S9A). We further identified the specific ECM components responsible for this remodeling. The major ECM components involved in regulating cell migration include proteins and polysaccharides. Gene Ontology analysis of ECM‐related DEGs highlighted a significant enrichment in collagen‐related biological processes and implicated the glycoprotein fibronectin (Figure 3F). In parallel, biochemical quantification of the decellularized matrix revealed a significant increase in total protein content, with no corresponding change in total polysaccharides (Figure S9B,C).

We further analyzed collagen and fibronectin in the ECM. Among the collagen components, types I, III, and IV, which are closely related to angiogenesis and cell migration, were specifically examined [41, 42]. Western blot and immunofluorescence analyses revealed a specific and significant upregulation of type I collagen at both the protein and secreted matrix levels, whereas levels of type III, type IV collagen, and fibronectin were unaffected (Figure 3G–I; Figure S9D). Collagen I is a primary component of the ECM, providing a structural framework that supports endothelial cell adhesion, migration, and tubular formation, which are essential for angiogenesis [43, 44]. To validate the causal role of collagen I, we used ethyl‐3,4‐dihydroxybenzoate (EDHB), an inhibitor of collagen I synthesis. EDHB treatment abrogated the rGO‐induced increase in ECM synthesis, as shown by SEM and total protein quantification (Figure 3J,K). Concurrently, EDHB co‐treatment significantly attenuated the pro‐migratory effect of rGO in the wound healing assay (Figure S9E). Taken together, these data demonstrate that rGO enhances SHED migration by promoting the synthesis of a collagen I‐rich ECM.

### 2.5 P4HA1‐Mediated Regulation Contributes to Collagen I Expression

**FIGURE 4:**
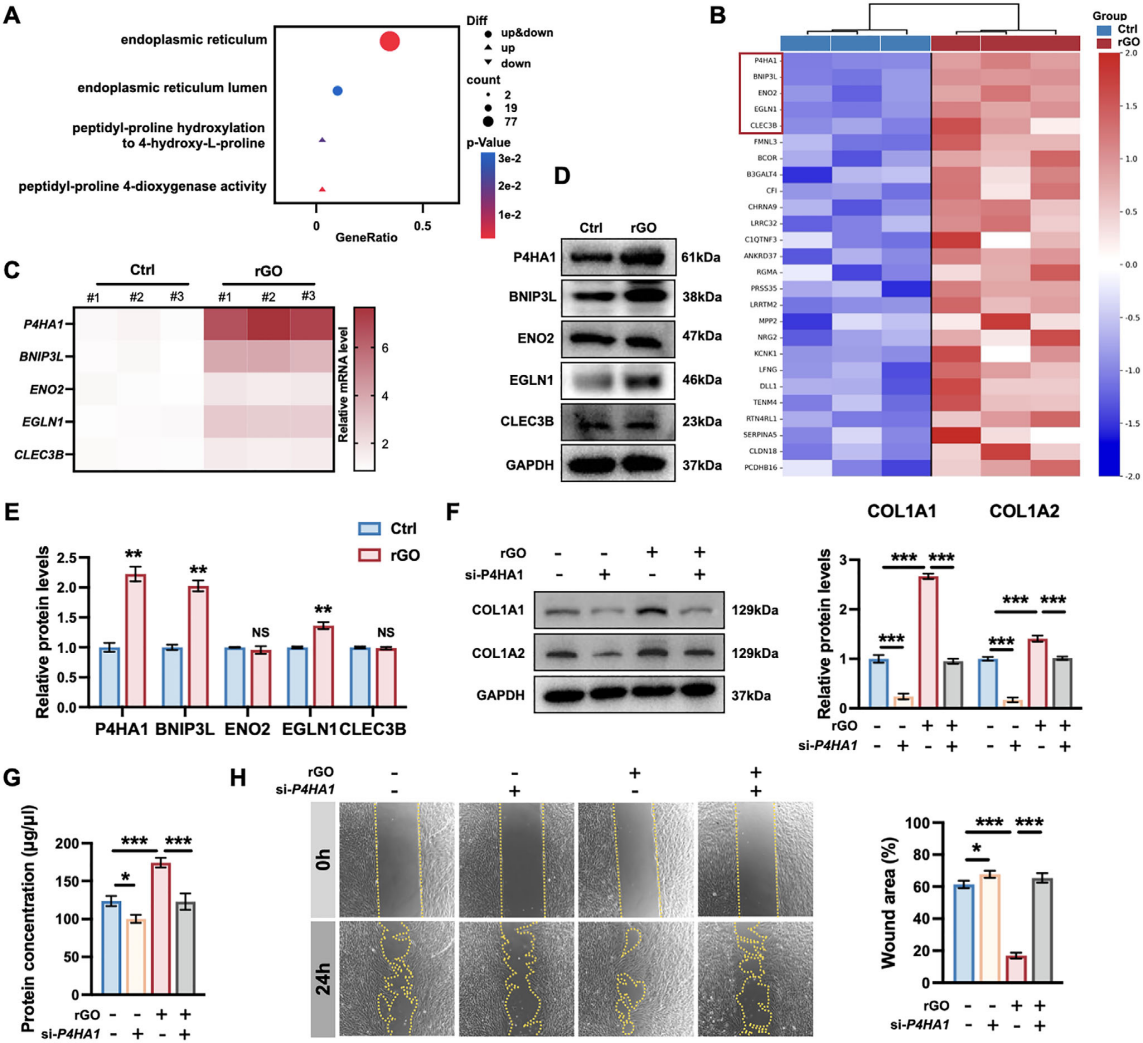
P4HA1‐mediated regulation contributes to collagen I expression. (A) GO enrichment analysis of collagen synthesis‐related DEGs, showing significant clustering in endoplasmic reticulum (ER)‐associated biological processes. (B) Heatmap displaying the differential expression of the top five ER‐related genes involved in collagen synthesis. (C) qRT‐PCR validation of the mRNA expression of the five candidate genes in SHEDs treated with rGO. (D,E) Western blot analysis and quantification of protein levels for the five candidates in SHEDs treated with rGO. (F–H) SHEDs were transfected with siRNA targeting *P4HA1* (si‐P4HA1) prior to treatment with rGO. (F) Western blot analysis of COL1A1 and COL1A2. (G) Quantification of total ECM protein content via BCA assay. (H) Representative images and quantification of the wound healing assay. Data are presented as mean ± SD (*n* = 3 per group). Statistical significance was determined using a two‐tailed unpaired *t*‐test for panel E and by a one‐way ANOVA with Tukey's *post hoc* test for panels F, G, and H. NS: no significance, ^*^ *p* < 0.05, ^***^ *p* < 0.001.

The increase in collagen I expression may arise from two processes: increased protein synthesis and/or reduced protein degradation. We first investigate the degradation pathway and confirmed that rGO treatment altered neither protein ubiquitination nor autophagic flux, thereby ruling out reduced degradation as the underlying mechanism (Figure S10A,B). We therefore turned our attention to the synthesis pathway, which broadly involves gene transcription followed by post‐translational processing within the endoplasmic reticulum (ER), where the constituent α1 (COL1A1) and α2 (COL1A2) protein chains are synthesized and assembled. However, our transcriptomic analysis revealed no significant changes in the mRNA levels corresponding to these chains upon rGO treatment, a finding that was further validated by qRT‐PCR analysis (Figure S10C). These findings indicate that the rGO‐induced increase in collagen I is not regulated at the transcriptional level, suggesting that it occurs via enhanced post‐translational synthesis within the ER. Consistently, GO enrichment analysis of collagen synthesis–related genes demonstrated significant clustering in ER‐associated processes (Figure 4A), where critical post‐translational modifications of procollagen, including hydroxylation and glycosylation, are known to occur [45].

Further analysis of ER‐related DEGs identified the top five upregulated candidates involved in procollagen processing: *P4HA1*, *BNIP3L*, *ENO2*, *EGLN1*, and *CLEC3B* (Figure 4B). While qRT‐PCR data confirmed the upregulation of all these genes at the mRNA level (Figure 4C; Figure S10D), only P4HA1, BNIP3L, and EGLN1 showed significant increases at the protein level after rGO treatment (Figure 4D,E). Therein, knockdown of *P4HA1*, in contrast to that of *BNIP3L* and *EGLN1*, blocked the rGO‐induced upregulation of COL1A1 and COL1A2 (Figure 4F; Figure S10E,F). Furthermore, *P4HA1* knockdown reversed the rGO‐mediated increase in total ECM protein deposition (Figure 4G) and abolished the enhancement in cell migration (Figure 4H). Collectively, these results identify P4HA1 as the critical mediator of rGO's pro‐migratory and ECM‐remodeling effects.

### 2.6 RELA Is the Upstream Transcription Factor of P4HA1

**FIGURE 5:**
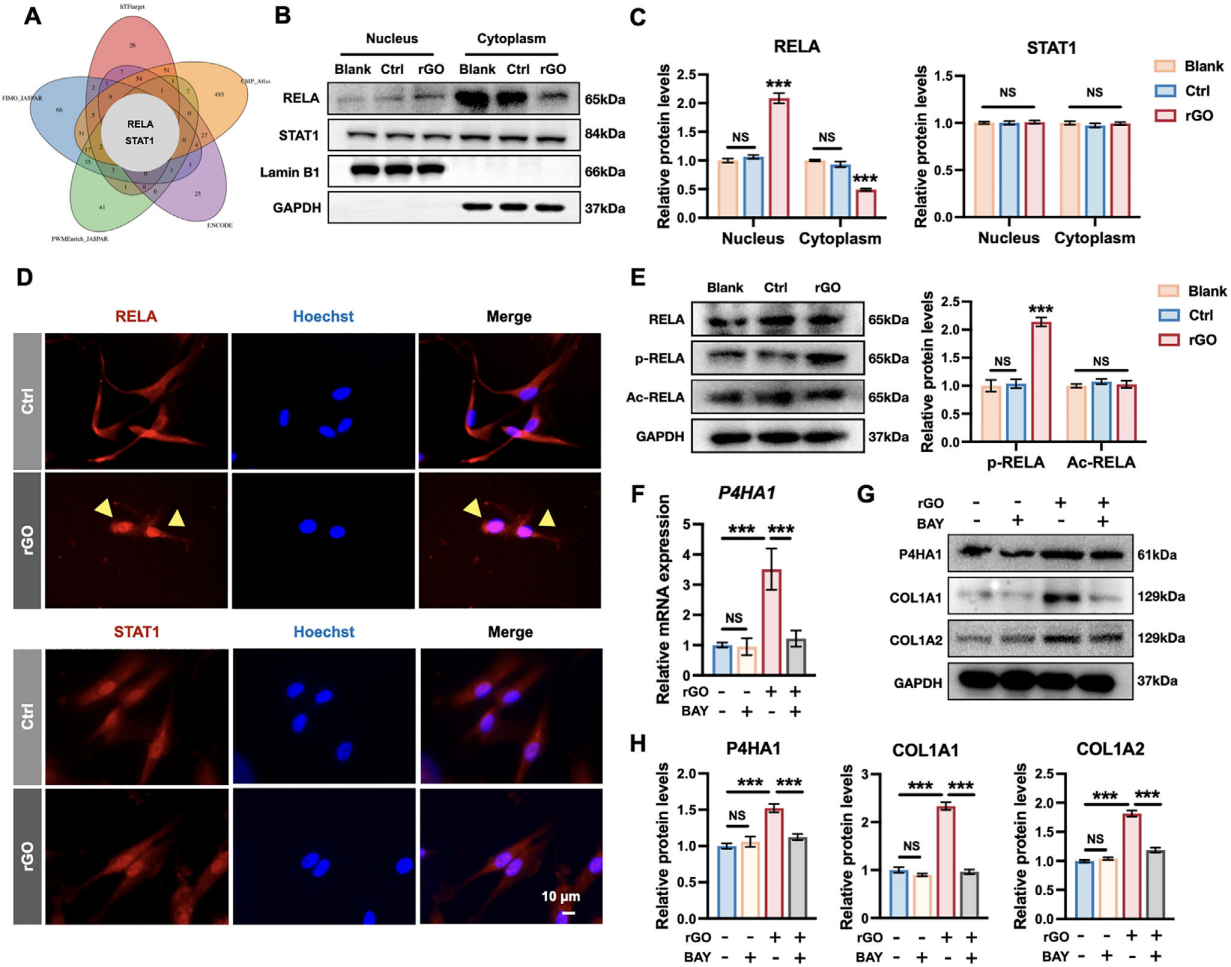
RELA is the upstream transcription factor of P4HA1. (A) Venn diagram showing the prediction of upstream transcription factors for *P4HA1* using integrated bioinformatic databases. (B,C) Western blot analysis and quantification of RELA and STAT1 in cytoplasmic and nuclear fractions of SHEDs treated with rGO. (D) Representative immunofluorescence images showing nuclear translocation of RELA (indicated by yellow arrowheads) in SHEDs upon rGO treatment. STAT1 localization remains unchanged. Scale bar, 10 µm. (E) Western blot analysis of total RELA, phosphorylated RELA (p‐RELA), and acetylated RELA (Ac‐RELA) in SHEDs treated with rGO. Changes in phosphorylated and acetylated protein levels were quantified after normalization to the corresponding total protein. (F–H) SHEDs were co‐treated with rGO and the RELA inhibitor BAY 11–7082 (BAY). (F) qRT‐PCR analysis of *P4HA1* mRNA expression. (G,H) Western blot analysis and quantification of P4HA1, COL1A1, and COL1A2. Data are presented as mean ± SD. (*n* = 3 per group). Statistical significance was determined using one‐way ANOVA with Tukey's *post hoc* test. NS: no significance, ^***^ *p* < 0.001.

Since P4HA1 was identified based on DEGs analysis, we speculated that the regulatory effect of rGO on P4HA1 was mediated through transcriptional regulation. The upstream transcription factor potentially responsible for P4HA1 upregulation was predicted by integrating multiple databases (hTFtarget, ChIP_Atlas, ENCODE, PWMEnrich_JASPAR and FIMO_JASPAR), and RELA together with STAT1 emerged as candidates (Figure 5A). To determine which TF directly regulates P4HA1, we assessed their nuclear translocation. Western blot analysis of nuclear and cytoplasmic fractions revealed a significant accumulation of RELA, but not STAT1, in the nucleus of rGO‐treated SHEDs, with a corresponding decrease level in the cytoplasm (Figure 5B,C). Immunofluorescence staining corroborated this finding, showing distinct nuclear localization of RELA specifically in the rGO group (Figure 5D). These findings indicate that rGO activates RELA and promotes its nuclear translocation, suggesting RELA as a key regulator of P4HA1.

Previous studies have suggested that RELA activation and subsequent nuclear translocation are primarily dependent on its phosphorylation [46, 47] and acetylation [48]. Our data showed that while its phosphorylated form (p‐RELA) was significantly elevated upon rGO treatment, the acetylation level was unaffected (Figure 5E), indicating that rGO activates RELA primarily via phosphorylation. To confirm the functional necessity of this event, we applied BAY 11–7082 (BAY), a specific inhibitor that blocks RELA phosphorylation [49, 50]. Co‐treatment with BAY effectively blocked the rGO‐induced phosphorylation of RELA. Crucially, this inhibition abrogated the upregulation of *P4HA1* at both the mRNA and protein levels. Consequently, the downstream increase in COL1A1, COL1A2, and total ECM protein deposition was also abolished (Figure 5F–H; Figure S11). These results establish a clear signaling axis where rGO induces RELA phosphorylation, leading to *P4HA1* transcription and subsequent collagen I‐driven ECM remodeling.

### 2.7 rGO‐Induced Actin Polymerization Activates RELA via FAK‐Src Signaling

**FIGURE 6:**
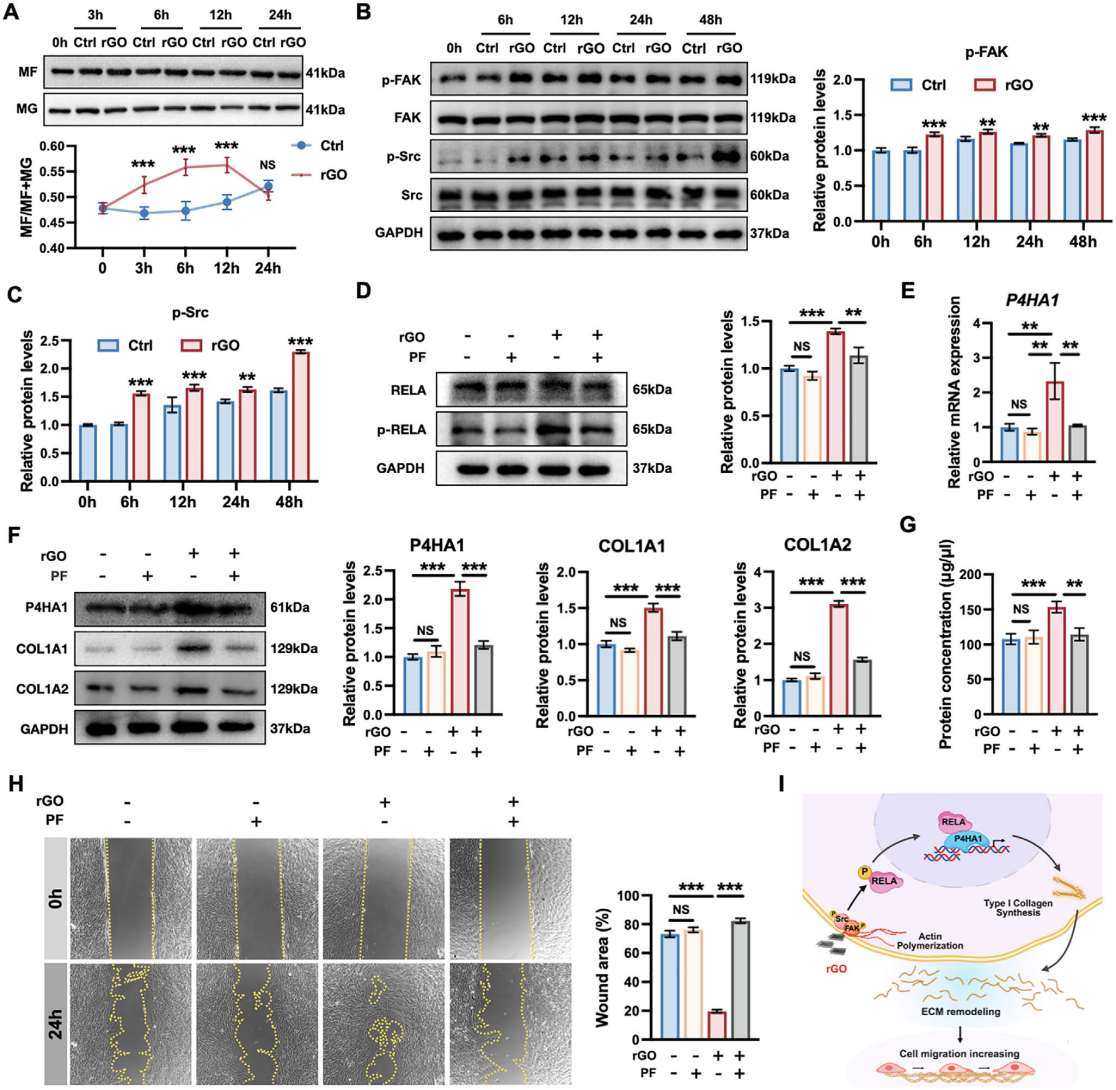
rGO‐induced actin polymerization activates RELA via focal adhesion kinase (FAK)‐Src signaling. (A) Western blot and quantification of the F‐actin/ (F‐actin + G‐actin) ratio in SHEDs treated with rGO for 3, 6, 12, and 24 h. (B,C) Western blot analysis and quantification of total and phosphorylated FAK and Src in SHEDs treated with rGO for 6, 12, 24, and 48 h. Changes in phosphorylated protein levels were quantified after normalization to the corresponding total protein. (D–H) SHEDs were co‐treated with rGO and the FAK inhibitor PF573228 (PF). (D) Western blot analysis of RELA phosphorylation. (E) qRT‐PCR analysis of *P4HA1* mRNA expression. (F) Western blot analysis of P4HA1, COL1A1, and COL1A2. (G) Quantification of total ECM protein content. (H) Representative images and quantification of the wound healing assay. Scale bar, 200 µm. (I) Schematic diagram illustrating the proposed signaling pathway. Data are presented as mean ± SD. (*n* = 3 per group). Statistical significance was determined using a two‐way analysis of variance (ANOVA) followed by Sidak's multiple comparisons test for panel A, B, C, and by a one‐way ANOVA with Tukey's *post hoc* test for panels D, E, F, G, and H. NS: no significance, ^**^ *p*<0.01 and ^***^ *p* < 0.001.

To elucidate the upstream signaling that triggers RELA phosphorylation, the potential regulatory pathways were investigated. Previous studies have demonstrated that RELA phosphorylation is predominantly regulated through two distinct mechanisms: either membrane‐associated components or cytoplasmic kinase cascades. Our group's prior work provides a foundation for this inquiry, having demonstrated that graphene‐based materials exhibit high affinity for membrane‐associated components and can modulate cellular behavior by altering actin dynamics [25, 51]. Time‐course analysis revealed that RELA phosphorylation occurred within 3 h of rGO exposure (Figure S12A). This timing correlated with our imaging data, which showed rGO's initial engagement with the cell membrane and subsequent modulation of the membrane‐associated actin cytoskeleton within a 1–3 h window (Figure 3B; Figure S8B). Previous studies have also reported that actin dynamics can activate RELA by regulating actin polymerization and depolymerization [52, 53]. Indeed, our data showed that rGO treatment induced enhanced actin polymerization, as evidenced by a significant increase in the F‐actin to G‐actin ratio (Figure 6A). Crucially, pharmacological inhibition of actin polymerization with cytochalasin D (Cyto‐D) abrogated rGO‐induced RELA phosphorylation, confirming that actin polymerization is a necessary upstream event (Figure S12B,C).

There are two candidate pathways involving mediating actin polymerization‐induced RELA phosphorylation: FAK/Src [54, 55] and PKC [56]. Western blot analysis revealed a selective and significant increase in the phosphorylation of FAK and Src in rGO‐treated cells (Figure 6B,C), whereas PKC phosphorylation level remained unchanged (Figure S12D). Moreover, inhibition of actin polymerization with Cyto‐D also prevented FAK/Src phosphorylation, positioning this axis downstream of actin polymerization (Figure S12E). To confirm the functional necessity of this axis, we employed a selective FAK inhibitor, PF573228 (PF) [57]. Inhibition of FAK completely abolished rGO‐induced RELA phosphorylation (Figure 6D), consequently abrogating the downstream molecular cascade including the upregulation of *P4HA1* and the subsequent deposition of collagen I and total ECM protein (Figure 6E–G). Functionally, this translated to a complete reversal of the pro‐angiogenic phenotype, as evidenced by suppressed cell migration, diminished expression of key angiogenic proteins, and impaired tube formation capacity (Figure 6H; Figure S13A,B).

The mechanism by which rGO activates the FAK/Src pathway can be attributed to its role as a potent mechanotransductive cue. It has been well‐documented that the distinct physicochemical properties of graphene family nanomaterials, including physical topography, oxygen‐containing functional groups, and surface hydrophobicity, can modulate the actin cytoskeleton [51]. Especially, rGO's topography, such as zigzag edges and the wrinkled surface, can impose physical stress and induce localized curvature on the cell membrane [58]. As shown in the characterization and immunofluorescence results, our rGO exhibited zigzag edges and a wrinkled surface, which enables it to affect both the cell membrane and the associated actin cytoskeleton. Subsequently, the increased cellular tension generated by these stressful actin fibers can be transmitted to focal adhesions, leading to the conformational activation and autophosphorylation of FAK, which in turn recruits and activates Src [59]. Thus, rGO is first recognized by the cell through physical interactions that reshape the cytoskeleton, and this mechanical signal is then transduced into a biochemical cascade via the FAK/Src pathway.

Taken together, these findings delineate a comprehensive molecular cascade: rGO induces actin polymerization that activates the FAK/Src signaling axis. This, in turn, drives RELA phosphorylation and nuclear translocation, leading to P4HA1 transcription, enhanced collagen I synthesis, and ultimately, promotion of cell migration and angiogenesis (Figure 6I).

### 2.8 In Vivo Validations of FAK‐Src/RELA/P4HA1 Pathway Using Prevascularized SHED‐Laden rGO@Hydrogel

**FIGURE 7:**
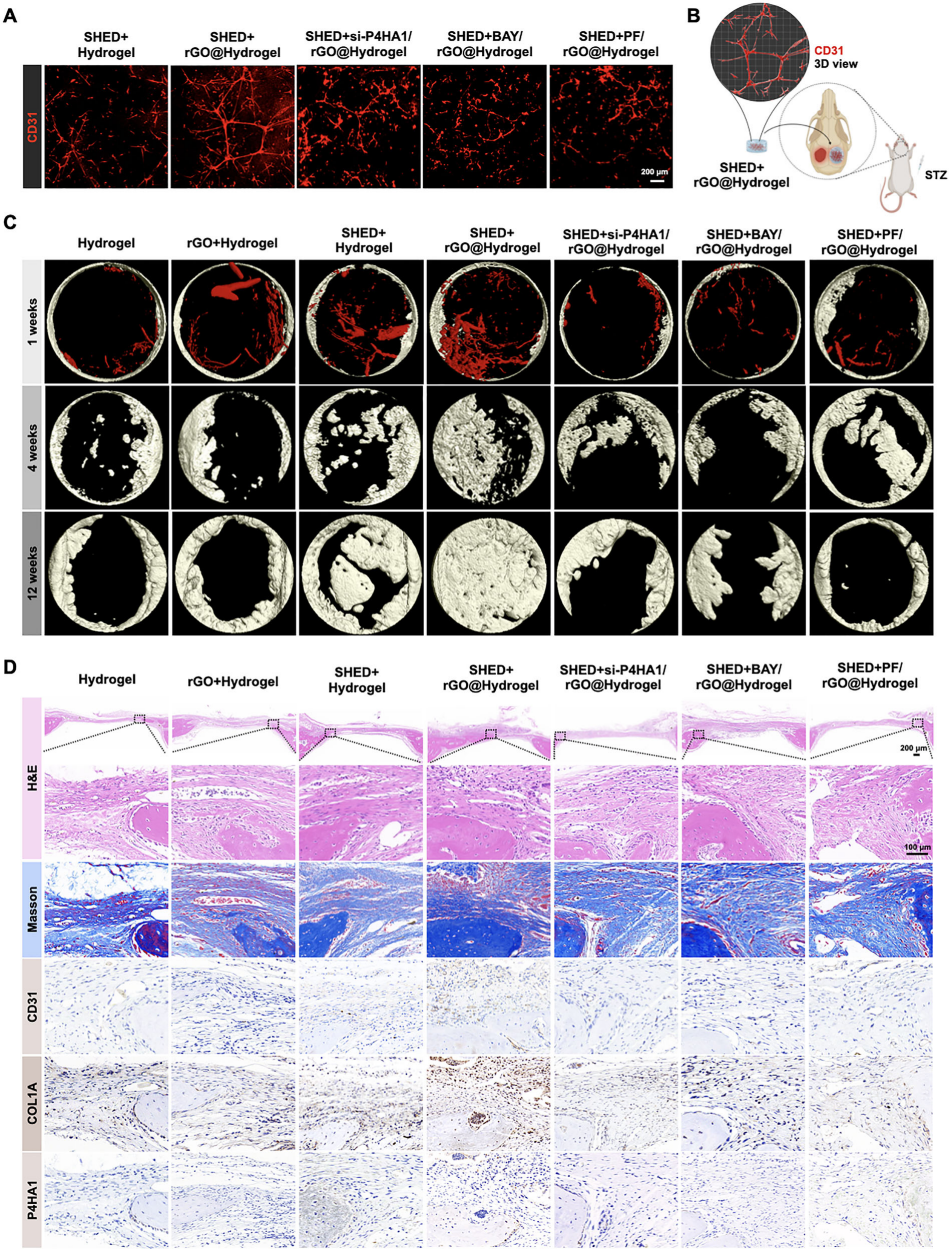
The FAK‐Src/RELA/P4HA1 axis is essential for the in vivo therapeutic efficacy of the prevascularized rGO@Hydrogel. SHED‐laden rGO@Hydrogels were treated with inhibitors (PF, BAY) or transfected with si‐P4HA1 prior to implantation. (A) Representative CD31 immunofluorescence images showing the extent of in vitro prevascularization in the different hydrogel constructs. Scale bar, 200 µm. (B) 3D reconstruction of CD31 immunofluorescence illustrating prevascularization. Schematic illustrating the implantation of the constructs into a diabetic rat calvarial defect model. (C) Representative micro‐CT 3D reconstructions of angiogenesis (1 week, top) and bone regeneration (4 week and 12 weeks, middle and bottom). (D) Representative histological (H&E, Masson) and immunohistochemical (CD31, COL1A, P4HA1) staining of the defect sites at 4 weeks post‐implantation. Scale bars, 200 µm (overview) and 100 µm (magnified inset).

To confirm that the FAK‐Src/RELA/P4HA1 signaling axis is essential for the in vivo therapeutic efficacy, we conducted a series of validation experiments. First, we confirmed that pharmacologically inhibiting FAK (with PF), RELA (with BAY), or P4HA1 (with si‐*P4HA1* transfected), significantly impaired the in vitro prevascularization of SHEDs within the rGO@Hydrogel, as evidenced by reduced CD31‐positive network formation (Figure 7A). We then implanted these pathway‐inhibited constructs into critical‐sized calvarial defects in diabetic rats (Figure 7B). This model was validated by its stable diabetic phenotype: hyperglycemia, polydipsia, and weight loss (Figure S14). In vivo fluorescence imaging of the ROS Brite 700 probe demonstrated significantly lower ROS levels at the defect site in the SHED+rGO@Hydrogel group compared to the SHED+Hydrogel control group, suggesting the potent in vivo antioxidant activity of rGO (Figure S15A). Micro‐CT angiography at 1‐week post‐implantation revealed robust neovascularization in the SHED+rGO@Hydrogel group, a therapeutic benefit that was largely abrogated in all inhibitor‐treated groups. Subsequent micro‐CT analysis at 4 and 12 weeks demonstrated the superior osteogenic potential of the SHED+rGO@Hydrogel, which achieved the highest bone volume, trabecular thickness, and bone mineral density. In contrast, bone regeneration was significantly compromised in all groups where the signaling axis was blocked (Figure 7C; Figure S15B–D).

Histological analyses were consistent with these findings. H&E and Masson's staining showed near‐complete defect bridging with well‐organized, mature bone and collagen matrix in the SHED+rGO@Hydrogel group, whereas the inhibitor groups displayed large residual defects and sparse, disorganized matrix. Crucially, immunohistochemical staining provided direct evidence for the activation of our proposed pathway in situ. The regenerated tissue in the SHED+rGO@Hydrogel group exhibited significantly higher expression of CD31, P4HA1, and COL1A, all of which were suppressed by the inhibitors and the si‐*P4HA1* transfection (Figure 7D; Figure S15E). Furthermore, re‐analysis of the diabetic beagle dog mandibular defects revealed significantly elevated levels of P4HA1 and COL1A in the SHED+rGO@Hydrogel group at both 4 and 12 weeks, confirming the activation of this pathway in a large‐animal model (Figure S16).

Collectively, these in vivo data demonstrate that the pro‐regenerative capacity of the prevascularized rGO@Hydrogel in diabetic bone defects is mechanistically dependent on the FAK‐Src/RELA/P4HA1 signaling axis. Beyond its immediate pro‐regenerative function, the long‐term fate of the rGO component is a critical consideration for clinical translation. AFM evidence of cell‐mediated exfoliation suggests a crucial first step toward its clearance [25]. This initial fragmentation creates smaller, more accessible structures, priming the otherwise degradation‐resistant rGO for subsequent, gradual clearance by host peroxidases (e.g., myeloperoxidase and eosinophil peroxidase) [60, 61, 62]. Although our systematic findings suggest it has no significant toxicity, longer‐term follow‐up studies are necessary to fully establish its clinical safety.

## 3 Conclusion

In summary, we have developed a prevascularized rGO‐SHED scaffold that promotes robust angiogenesis and bone regeneration in a clinically challenging large‐animal diabetic model, underscoring its significant translational potential. More importantly, we have uncovered the underlying mechanobiological principle: rGO acts as a direct physical cue to guide SHEDs in actively remodeling their own extracellular matrix into a pro‐angiogenic niche. It was achieved through the modulation of cytoskeletal dynamics and the activation of the FAK‐Src/RELA‐P4HA1 signaling axis, which drives targeted collagen synthesis. This study therefore establishes material‐guided, cell‐mediated ECM engineering as a potent strategy to overcome the regenerative barriers of pathological microenvironments.

## 4 Methods

### 4.1 Green Synthesis of rGO

*R. rosea* (Tongrentang, China) was washed with double‐distilled water, then dried and ground at 50°C. The product was sieved and dissolved in 95% ethanol. The mixture underwent extractions in a 70°C water bath for 3 times, each lasting for 30 min. After each extraction, the solution was filtered. The three filtrates were mixed and concentrated to half of their original volume through evaporation at 50°C. Graphene oxide nanosheets (Nanoinnova, Spain), were added to the *R. rosea* extract. The mixture was stirred at a temperature of 40°C for 8 h using a magneto‐stirrer heater (MS‐H‐S, DLAB, China), following by 80°C for 1 h. Then, the mixture was filtered and washed with deionized water for 3 times to obtain rGO. The size and morphological characteristics of rGO were examined through AFM (Dimension Edge, Bruker, Germany). The surface chemical composition of GO was analyzed using an x‐ray photoelectron spectrometer (XPS, K‐Alpha, Thermo Fisher Scientific, USA).

### 4.2 Cell Culture and Endothelial Differentiation Induction

SHEDs were isolated from teeth as previously reported [63] and approved by the Ethics Committee of the Stomatological Hospital of Southern Medical University (approval number: NYKQ‐EC‐[2024]‐41). Briefly, dental pulp tissue was digested with collagenase (C1‐28‐100MG, Sigma, USA), processed into a single‐cell suspension, and cultured in MEM α medium (12571063, Thermo Fisher Scientific, USA) with 10% FBS (C0235, Invitrogen, USA) and 1% penicillin‐streptomycin (15140122, Invitrogen, USA). For endothelial differentiation, cells were cultured in EGM‐2 medium (CC3162, Lonza, USA), and differentiation was confirmed by western blotting for endothelial markers (CD31, KDR, vWF) on days 1, 3, 5, and 7.

### 4.3 Cell Viability Determination

Following 5 days of endothelial differentiation, SHEDs were treated with various concentrations of rGO (0, 0.5, 1, 2.5, 5, 10, 25, 50, 100 µg/mL) for 1, 3, and 5 additional days, and their viability was subsequently assessed using a CCK‐8 assay (C0039, Beyotime, China) that involved washing the cells with PBS and incubating them with the diluted solution in the dark. The resultant solution was transferred to a fresh 96‐well plate with the absorbance measured at 450 nm using a multimode microplate reader (BioTek Synergy H1, Agilent, USA).

### 4.4 Western Blot

Proteins were extracted using the lysis buffer (P0013, Beyotime, China) containing phosphatase (HY‐K0011, MCE, USA) and protease inhibitors (HY‐K0010, MCE, USA). Protein concentrations were detected by the Pierce BCA protein assay kit (23227, Thermo Fisher, USA). Proteins were parted by SDS‐PAGE gel and moved to PVDF membranes (IPVH00010, Millipore, USA). After blocking, membranes were incubated with primary antibodies, followed by appropriate secondary antibodies. Protein bands were visualized using an ECL detection kit (WBLKS0500, Merck Millipore, USA) and imaged with a Tanon 5200 automatic chemiluminescence imaging system (Tanon, China). Protein expression levels were quantified using ImageJ software (National Institutes of Health, USA). Information about the antibodies used in this study is provided in Table S1.

### 4.5 Tube Formation Assay

Endothelial‐induced SHEDs were treated with rGO at concentrations of 1, 5, and 10 µg/mL for 1, 3, 5, and 7 d. The cells were later harvested and seeded onto 96‐well plates coated with Matrigel. The formation of capillary‐like structures was observed and captured using a microscope (DM2500, Leica, Germany). Quantitative analysis of the angiogenic potential including the total mesh area, total tube nodes, master segment length, and quantified the number of master junctions was performed using ImageJ software.

### 4.6 Preparation and Characterization of rGO@Hydrogel

The rGO solution was sonicated to ensure its uniform dispersion. Then it was mixed with Matrigel (354230, Corning, USA) to reach a final concentration of 5 µg/mL and placed for 30 min to form the rGO@Hydrogel. The rGO@Hydrogel surface morphology was examined using SEM (JSM‐7500F, JEOL, Japan) at 20 kV accelerating voltage. The degradation capability was evaluated by immersing pre‐weighed hydrogel samples in either PBS or cell culture medium at 37°C and tracking their weight change at predetermined time points of 3, 7, 14, 21 and 28 d. The degradation rate was calculated based on the proportion of hydrogel mass remaining relative to the initial weight.

### 4.7 The Prevascularization of rGO+SHED@Hydrogel

After endothelial differentiated for 5 d, SHEDs were harvested and encapsulated in rGO@Hydrogel at a final concentration of 1 × 10^6^ cells/mL and then cultured for 3 days. Angiogenic sprouting and tube formation, was characterized by immunostaining for CD31 and visualized using laser scanning confocal microscopy (STELLARIS 5, Leica, Germany). Random positions were imaged by a z‐stack imaging volume of 500 µm × 500 µm × 80 µm, according to the published literature [64]. Data were reconstructed using Imaris software (Imaris 10.2, Oxford instrument, UK).

### 4.8 In Vivo Study

#### 4.8.1 Animal Model

All procedures involving beagle dogs were approved by the Animal Ethics Committee of Guangdong Medical Laboratory Animal Center (approval number: B202412‐1). Diabetes was induced by consecutive intravenous injections of streptozotocin (STZ) at a dose of 30 mg/kg for three consecutive days. Following the final injection, the successful establishment of the diabetic model was confirmed by a sustained elevated fasting blood glucose level, increased water intake, and weight loss. In accordance with the 3Rs principles for animal ethics, two beagle dogs were utilized for each designated time point. Following general anesthesia with Zoletil50 (Virbac, France), three standardized circular bone defects (8 mm in diameter) were created on each side of the mandibular body. A total of nine defects were randomly allocated to three experimental groups: Hydrogel group, SHED‐laden hydrogels (SHED+Hydrogel group), and SHED‐laden rGO@Hydrogel constructs (SHED+rGO@Hydrogel group). Each group consisted of three defects (*n* = 3) for statistical analysis.

All rat experiments were conducted in accordance with protocols approved by the Research Ethics Committee of Guangzhou Fangwei Biotechnology Co., Ltd. (approval number: FW2025050701). Diabetes was induced in SD rats (male, 200–220 g) by intraperitoneal injection of STZ (65 mg/kg) [65]. One week later, rats were screened for diabetes, and only those with fasting blood glucose levels consistently above 16.7 mmol/L, accompanied by significant body weight loss and polydipsia, were included in the subsequent surgical procedures. For the establishment of the calvarial defect model, rats were anesthetized with isoflurane (R510‐22‐16, RWD, China), and a midline scalp incision was made to expose the calvaria. Bilateral full‐thickness circular defects (5 mm in diameter) were created symmetrically on either side of the sagittal suture using a trephine drill. The two calvarial defects in each rat served as paired experimental and control sites. Based on the scaffold composition and treatment, the defects were randomly allocated to one of seven groups: Hydrogel, rGO+Hydrogel, SHED+Hydrogel, SHED+rGO@Hydrogel, SHED+rGO/si‐P4HA1@Hydrogel, SHED+rGO/BAY@Hydrogel, and SHED+rGO/PF@Hydrogel. Inhibitors BAY 11–7082 (HY‐13453) and PF‐573228 (HY‐10461R) were purchased from MedChemExpress (USA). Angiogenesis was assessed 1‐week post‐implantation, while new bone formation was evaluated at 4 and 12 weeks. For statistical analysis, three independent defect sites (*n* = 3) were analyzed for each group at each of the specified time points.

#### 4.8.2 Micro‐CT

Micro‐CT was used to visualize angiogenesis and bone formation. For angiogenesis evaluation, the rats were perfused heparinized saline and 4% paraformaldehyde solution, followed by Microfil (MV‐112, Flow Tech, USA) at 1 week [66]. For bone evaluation, the rats were perfused 4% paraformaldehyde solution. 3D reconstruction of the vascular and bone was performed based on the micro‐CT data set. The vascular volume fraction (vascular BV/TV), vascular number, BV/TV, Tb. Th and BMD were directly analyzed according to the 3D reconstruction images.

#### 4.8.3 Hematoxylin and Eosin Staining (H&E) and Masson Staining

Skull and mandible samples were decalcified in 10% EDTA for 12 weeks, then embedded in paraffin and subsequently prepared into sections. Paraffin sections were first deparaffinized, then stained with hematoxylin and eosin (C0105, Beyotime, China). After staining, the sections were sealed with neutral resin. For masson staining, the sections were then stained with Modified Masson's Trichrome Stain Kit (G1346‐8, Solarbio, China), according to the manufacturer protocols. After staining, the sections were sealed with neutral resin.

#### 4.8.4 Immunohistochemistry

Paraffin sections were first deparaffinized and heat‐treated in citrate buffer (ZLI‐9065, ZSGB‐BIO, China) for antigen retrieval. The sections were then blocked with 5% goat serum (AR0009, Boster Biological Technology, USA) to reduce non‐specific binding. Subsequently, the sections were incubated overnight at 4°C with primary antibodies. After washing, the sections were treated with an anti‐rabbit IgG SABC‐HRP kit (P0615, Beyotime, China). The sections were finally stained via a diaminobenzidine kit (ZLI‐9018, ZSGB‐BIO, China) and sealed with neutral resin.

### 4.9 RNA Extraction and qRT‐PCR Analysis

Total RNA extraction from cellular samples was performed using TRIZOL reagent (AG21101, Accurate Biology, China). Subsequently, cDNA was obtained using RT Premix (AG11706, Accurate Biology, China). qRT‐PCR was conducted with SYBR Green Master Mix (AG11701, Accurate Biology, China) using a LightCycler 96 instrument (Roche, Switzerland). The relative gene expression levels were calculated using the 2^−ΔΔ^Ct method. Information about the primer pair used in this study is provided in Table S2.

### 4.10 Cell Migration Assay

Differentiation‐induced SHEDs were treated with rGO for 3 or 5 days, harvested, and reseeded into a pre‐insert wound‐healing culture system (ibidi, 81176, Germany). Upon reaching 100% confluence, the inserts were removed to create a linear wound in each cell monolayer. The healing area, indicating cell migration, was subsequently quantified using ImageJ software.

### 4.11 Immunofluorescence and Fluorescent Probe Staining

After washing with PBS, cells were first labeled with the lipophilic fluorescent dye DiO (C1993S, Beyotime, China) according to the manufacturer's instructions to visualize the cell membrane. Subsequently, cells were fixed by 4% paraformaldehyde solution, and permeabilized by 0.1% triton (P0096, Beyotime, China), and blocked with 5% BSA solution. The cells were then incubated with primary antibodies, followed by the corresponding fluorescent secondary antibodies, phalloidin (ab176757, Abcam, UK), and Hoechst (C1027, Beyotime, China). Fluorescent images were captured using a confocal microscope.

### 4.12 RNA‐seq

RNA was isolated from cell samples that had been treated with either 0 or 5 µg/mL of rGO for 2 d, using TRIZOL reagent (AG21101, Accurate Biology, China). The extracted RNA was then assessed for both quantity and quality. Sequencing libraries were subsequently generated using the Hieff NGS Ultima Dual‐mode mRNA Library Prep Kit (12301, Yeasen Biotechnology, China). Bioinformatic analysis was performed on the GenePlus Cloud platform (GenePlus, China). Raw reads were first filtered to remove low‐quality sequences and adapter contaminants, and the clean reads were subsequently aligned to the human reference genome (GRCh38/hg38). Differential expression analysis was performed using the DESeq2 package. Genes meeting the criteria of an adjusted p‐value (padj) < 0.05 and an absolute log2(Fold Change) > 1 were identified as DEGs. Finally, functional and pathway enrichment analyses for these DEGs were conducted based on the GO and Kyoto Encyclopedia of Genes and Genomes (KEGG) databases.

### 4.13 Preparation and Characterization of dECM

SHED‐dECM was prepared using a previously reported decellularization protocol [67]. Briefly, cells were treated with 0.5% Triton X‐100 and 20 mm NH_4_OH for 5 min at 37°C. Subsequently, residual nucleic acids were removed by a 2 h digestion at 37°C with DNase (50 U/mL, D7076, Beyotime, China) and RNase (50 µg/mL, ST577, Beyotime, China). For ultrastructural characterization, dECM samples were fixed with 2.5% glutaraldehyde at 4°C overnight, dehydrated through a graded ethanol series, critical‐point dried, gold sputter‐coated, and examined by SEM (Sigma 300, Zeiss, German). Total protein content of dECM was quantified using a BCA protein assay according to the manufacturer's instructions. Absorbance was measured at 562 nm, and protein concentration was calculated based on a bovine serum albumin standard curve.

### 4.14 Separation of Nucleus and Cytoplasmic Proteins

Nucleus and cytoplasmic protein extraction kits (WLA020, Wanleibio, China) were used. For the cytoplasmic protein, add the cytoplasmic protein extraction reagent and mix thoroughly. Centrifuge the mixture at 12 000 rpm. The resulting supernatant contains the cytoplasmic proteins. Then, add the nuclear protein extraction reagent to the remaining pellet and mix well. Centrifuge this mixture again at 12 000 rpm. The supernatant obtained from this second centrifugation contains the nuclear proteins. The protein expression level was then analyzed by western blot.

### 4.15 Isolation of Polymerized and Depolymerized Actin

Polymerized (F‐actin) and depolymerized (globular actin, G‐actin) actin fractions were isolated using an established actin fractionation protocol [68, 69]. Briefly, cells were lysed on ice in actin stabilization buffer (0.1 m PIPES, 30% glycerol, 5% DMSO, 1 mm MgSO_4_, 1 mm EGTA, 1% TritonX‐100, 1 mm ATP, and protease inhibitor, pH 6.9) and centrifuged at 16 000 × g for 75 min. The supernatant containing G‐actin was collected, while the pellet enriched in F‐actin was resuspended in actin depolymerization buffer (0.1 m PIPES, 1 mm MgSO_4_, 10 mm CaCl_2_, and 5 µm Cyto‐D, pH 6.9). Equal amounts of the two fractions were separated by SDS–PAGE and analyzed by western blotting using an anti‐β‐actin antibody. The F/G‐actin ratio was calculated by densitometric analysis.

### 4.16 Small Interfering RNA (si‐RNA) Transfection

siRNA targeting *P4HA1*, *BNIP3L*, and *EGLN1* (Tsingke, China), together with a non‐targeting control siRNA were used for gene silencing. Cells were seeded in culture plates and transfected at approximately 60%–70% confluence using Lipofectamine 3000 (L3000015, Invitrogen, USA) according to the manufacturer's instructions. Briefly, siRNA and transfection reagent were diluted separately in Opti‐MEM and then mixed to form siRNA–lipid complexes, which were added to the cells. After 6 h of incubation, the medium was replaced with fresh complete culture medium. Cells were harvested 48 h post‐transfection for subsequent analyses.

### 4.17 Statistical Analysis

Statistical analysis was conducted by GraphPad Prism10 (GraphPad Software, USA). All data are described as mean ± standard deviation (SD). Each experiment was conducted with at least three independent biological replicates. For comparisons between two groups, a two‐tailed unpaired *t*‐test was employed. For multiple group comparisons with a single factor, one‐way analysis of variance (ANOVA) was utilized, followed by Tukey's post hoc test. For multiple group comparisons with two independent factors, two‐way ANOVA was utilized, followed by Sidak's multiple comparisons test. Statistical significance was defined as *p* < 0.05.

## Funding

This Project was supported by grants from the National Natural Science Foundation of China (82271025, 82571055); Guangdong Provincial Association for Science and Technology Youth Science and Technology Talent Cultivation Program(2025QPRC17); Young Talent Support Project of Guangzhou Association for Science and Technology (QT2024‐031), and Science research cultivation program of stomatological hospital, Southern Medical University (RC202403).

## Ethics Statement

Experiments using human stem cells from exfoliated deciduous teeth (SHED) were approved by the Ethics Committee of the Stomatological Hospital of Southern Medical University (approval number: NYKQ‐EC‐[2024]‐41). All animal experiments were conducted in accordance with the National Research Council's Guide for the Care and Use of Laboratory Animals. Experiments involving beagle dogs were approved by the Animal Ethics Committee of Guangdong Medical Laboratory Animal Center (approval number: B202412‐1), while procedures involving rats were approved by the Research Ethics Committee of Guangzhou Fangwei Biotechnology Co., Ltd. (approval number: FW2025050701).

## Consent

All authors agree to the publication.

## Conflicts of Interest

The authors declare no conflicts of interest.

## Supporting information




**Supporting File**: advs75524‐sup‐0001‐SuppMat.docx.

## Data Availability

The datasets used and/or analyzed during the current study are available from the corresponding author upon reasonable request.
