# Supplementary material for: High‐Performance Prevascularized SHED‐Laden rGO@Hydrogel Achieves Optimized Diabetic Bone Defect Repair
Source: Adv Sci (Weinh). 2026 May 7;13(42):e75524. doi: 10.1002/advs.75524 (PMC13335533; doi:10.1002/advs.75524)
Supplement: Supplementary file 1 — Supporting File: advs75524‐sup‐0001‐SuppMat.docx. [file ADVS-13-e75524-s001.docx]

**High-performance Prevascularized SHED-laden rGO@Hydrogel Achieves Optimized Diabetic Bone Defect Repair**

Can Zhang^1, #^, Yiyuan Kang^#, *,1,2^, Shulin Lai^1^, Chunyi Wang^1^, Guixin He^1^, Kehui Jian^1^, Suhan Yin^1^, Xiner Tan^1^, Xinru Zhou^1^, Wenjing Liu^1^, Fujian Zhao^1^, Jia Liu^*,1^, Longquan Shao^*,1,2^

^1^ Stomatological Hospital, Southern Medical University, Guangzhou 510280, China

^2^ Guangdong Provincial Key Laboratory of Construction and Detection in Tissue Engineering, Guangzhou 510515, China

^#^These authors contributed equally

^*^Corresponding author: shaolongquan@smu.edu.cn (Longquan Shao); kangyiyuan@smu.edu.cn (Yiyuan Kang); liujia1988@smu.edu.cn (Jia Liu)

**1 Methods**

**1.1 Characterization of rGO**

Hydrazine-synthesized reduced graphene oxide (H-rGO) was purchased from Aladdin Bio-Chem Technology Co., Ltd. (R487603, China). The functional groups of *R. rosea*- rGO and graphene oxide were characterized by FT-IR (FT-IR Frontier, PerkinElmer, USA). The size and morphology of rGO were characterized by transmission electron microscopy (TEM, Talos F200S G2 S, Thermo Fisher Scientific, USA). Raman spectroscopy of GO was performed with a Raman microscope (LabRAM HR Evolution, HORIBA Scientific, France). The crystalline structure of rGO was characterized by X-ray diffraction (XRD, SmartLab-9kw, Rigaku, Japan).

**1.2 Extracellular reactive oxygen species (ROS) scavenging assay**

Extracellular ROS scavenging by rGO was quantified using the Amplex® Red Hydrogen Peroxide/Peroxidase Assay Kit (A22188, Thermo Fisher Scientific, USA). SHEDs were seeded in 96-well plates (2 × 10⁴ cells/well) and cultured overnight. The following day, cells were treated with 100 μM H₂O₂ and rGO (1, 5, 10 µg/mL) in phenol red-free MEM α medium for 2 h. Subsequently, 50 μL of culture supernatant was collected and mixed with 50 μL of the Amplex Red/HRP working solution. After a 30-min incubation in the dark, fluorescence was measured (Ex/Em: ~560/590 nm) to determine the remaining H₂O₂. All values were normalized to the H₂O₂-only control group.

**1.3 Flow cytometric identification of SHEDs as mesenchymal stem cells (MSCs)**

SHEDs were identified as MSCs by flow cytometry. Briefly, single-cell suspensions were incubated with fluorochrome-conjugated primary antibodies against human CD73, CD90, CD105 (positive markers), and CD34, CD45, HLA-DR (negative markers) for 30 min at 4°C in the dark. Unstained cells were used as a negative control. After washing with PBS, cells were analyzed on a flow cytometer (DxFLEX, Beckman, USA).

**1.4 Live/dead cell staining**

Cell viability was assessed using a Live/Dead Cell Staining Kit (BB-4126, BestBio, China) according to the manufacturer’s instructions. Hydrogel-encapsulated cells were washed with PBS and incubated with a working solution containing Calcein-AM (for live cells, green fluorescence) and Propidium Iodide (PI) (for dead cells, red fluorescence) for 15 min in the dark. Samples were then visualized by fluorescence microscopy, and representative images were captured.

**1.5 Cell proliferation assay**

Differentiation-induced SHEDs were treated with rGO for 1, 3, or 5 d. Then cell proliferation analysis was conducted using the 5-ethynyl-2’-deoxyuridine (EdU) cell proliferation kit (C0071S, Beyotime, China) according to the manufacture’s instruction. SHEDs, treated as indicated, were incubated with 10 μM EdU for 2 h. After fixation, permeabilization, and a Click-iT reaction to label incorporated EdU, samples were processed for analysis. For imaging, cells were counterstained with Hoechst 33342 and visualized by confocal microscopy. For quantitative analysis, total fluorescence intensity was measured using a multimode microplate reader.

**1.6 Dimethylmethylene blue (DMMB) assay**

The glycosaminoglycan (GAG) content in decellularized extracellular matrix (dECM) samples was quantified using a DMMB assay kit (GMS19239.1 GENMED, USA). Briefly, dECM samples were digested overnight at 60°C in papain digestion buffer (0.1 M sodium phosphate, 10 mM EDTA, 10 mM L-cysteine, pH 6.5, containing 125 µg/mL papain). After digestion, the samples were centrifuged, and the supernatants were incubated with the DMMB dye solution. The absorbance was measured at 525 nm using a microplate reader, and GAG content was calculated based on a standard curve generated with chondroitin sulfate.

**1.7 Co-IP analysis**

Cells were lysed on ice in binding buffer (50 mM Tris, 150 mM NaCl, and 0.1% TritonX-100, pH 7.5) supplemented with a protease inhibitor cocktail. The resulting lysates were incubated with the indicated primary antibodies and magnetic beads at 4 °C overnight. After incubation, the beads were collected using a magnetic separator and extensively washed with wash buffer of the same composition. The immunocomplexes were then eluted by adding 2× loading buffer and heating at 95 °C for 10 min. Following centrifugation to remove the beads, the supernatants containing antigen–antibody complexes were subjected to Western blot analysis. Whole-cell lysates were analyzed in parallel as input controls.

**1.8 *In vivo* ROS Detection in Skull Defects**

Four weeks post-surgery, rats with skull defects were used for in vivo detection of ROS using the ROS Brite^TM^ 700 probe (16004, AAT Bioquest, USA). After anesthetizing rats with isoflurane, 100 μM ROS Brite^TM^ 700 (in Hanks with 20 mM HHBS) was injected directly into the cranial region. Bioluminescence images, indicative of ROS production within the defect area, were then acquired using an in vivo imaging system (IVIS Lumina III, Revvity, USA).

**2 Results**


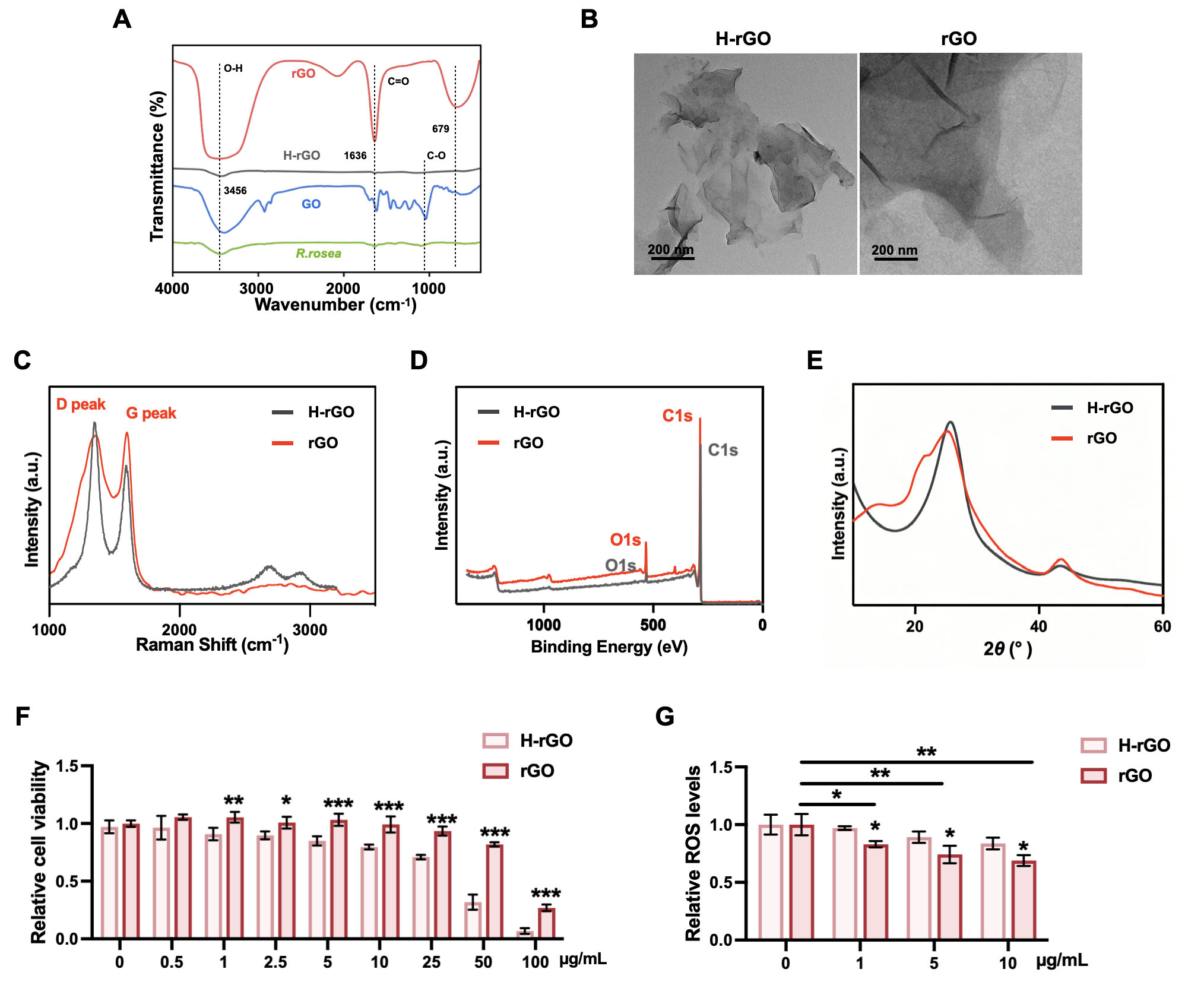


**Fig. S1 Characterization of rGO**

Physicochemical characterization of rGO using (A) FT-IR spectra characterizing the chemical bonds and functional groups of graphene oxide, H-rGO, rGO and *R. rosea*. (B) TEM image showing the sheet-like morphology of H-rGO and R-rGO. (C) Raman spectrum. (D) XPS spectra. (E) XRD spectra. Biological characterization using (F) CCK-8 showing the viability of SHEDs after treatment with different concentrations of or H-rGO for 1 day. (G) Quantification of ROS levels in the culture supernatant from H_2_O_2_-challenged SHEDs treated with rGO. Data in (F) and (G) are presented as mean ± SD (n=3 per group). Statistical significance was determined using a two-way analysis of variance (ANOVA) followed by Sidak’s multiple comparisons test for panel F and G. **P* < 0.05, ***P* < 0.01, and ****P* < 0.001.


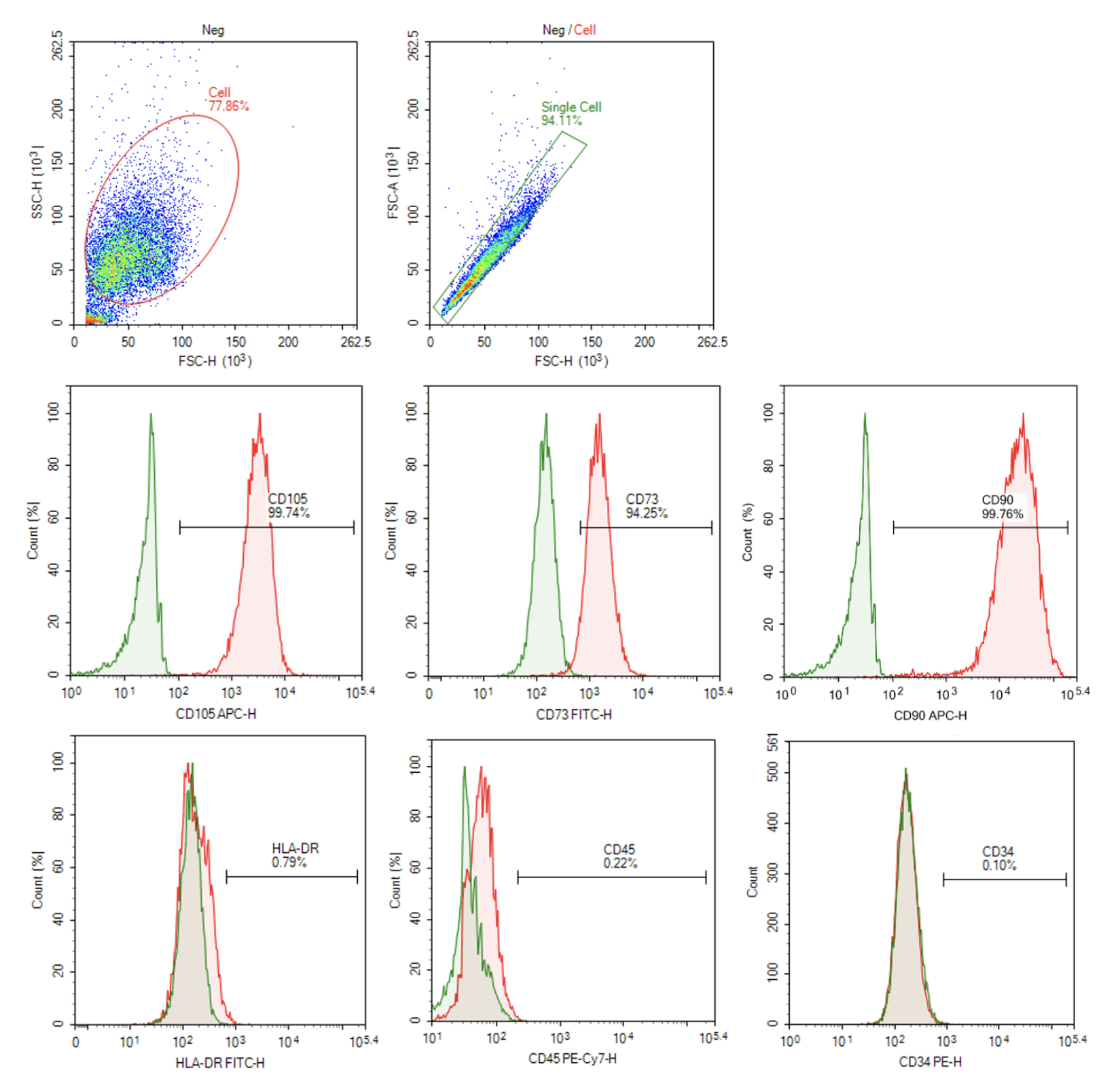


**Fig. S2 cytometric identification of SHEDs as mesenchymal stem cells**

Representative histograms showing high expression of positive MSC markers (CD105, CD73, CD90) and low to negligible expression of negative hematopoietic/endothelial markers (CD34, CD45, HLA-DR) in cultured SHEDs.


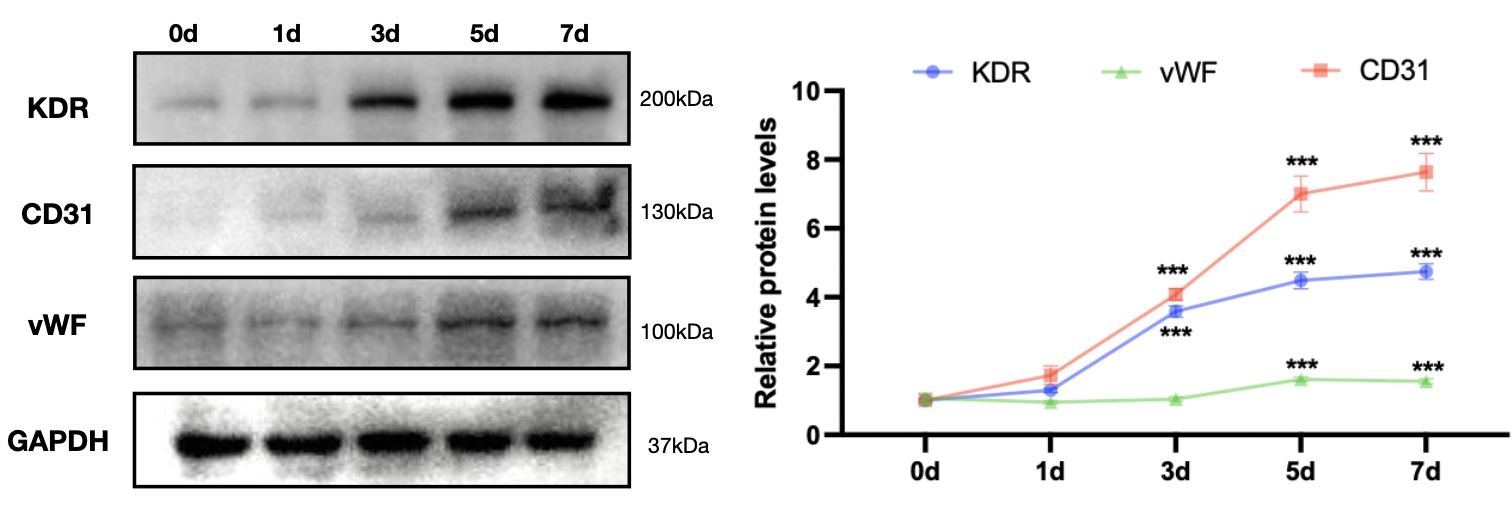

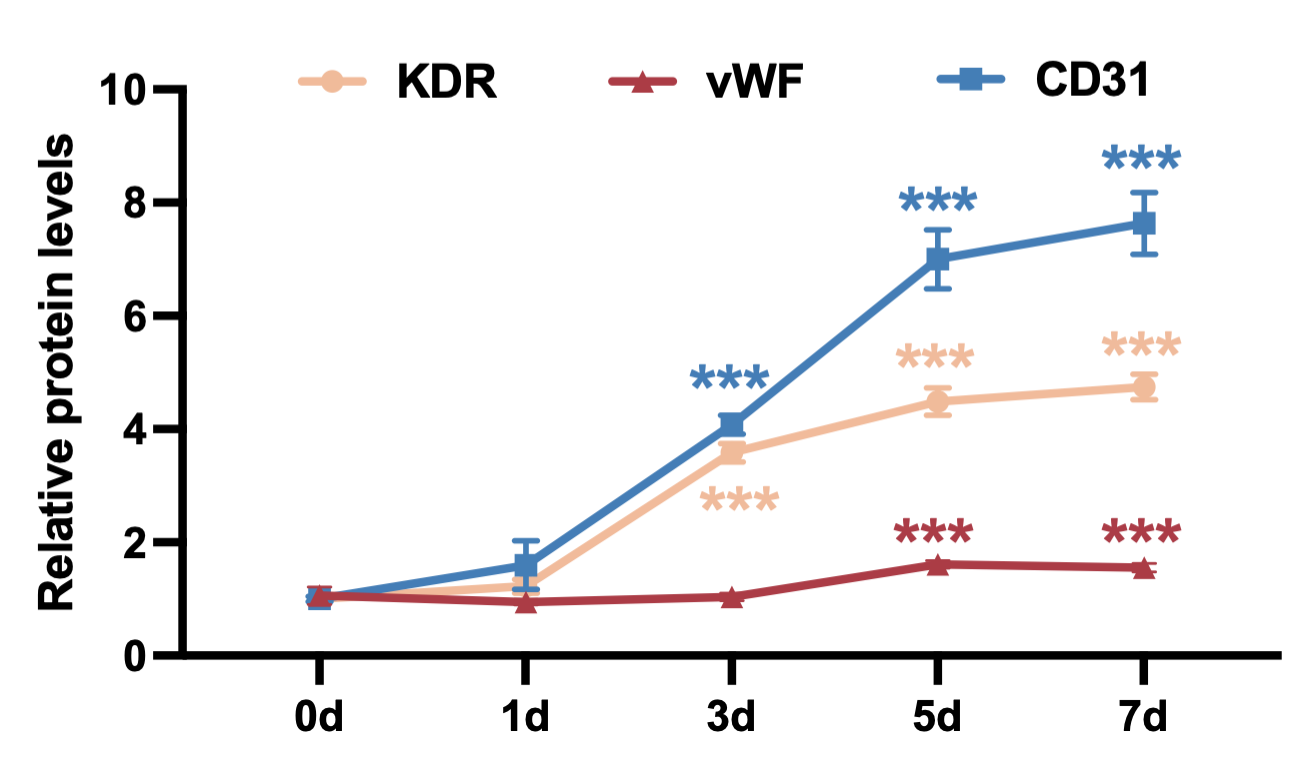


**Fig. S3** **Time-course analysis of endothelial marker expression in SHEDs**

Western blot analysis showing the protein levels of endothelial differentiation-related markers (KDR, vWF, CD31) in SHEDs cultured in endothelial growth medium (EGM2) for 0, 1, 3, 5, and 7 d. n=3 per group. Statistical significance was determined using a one-way ANOVA with Tukey’s *post hoc* test. ****P* < 0.001.


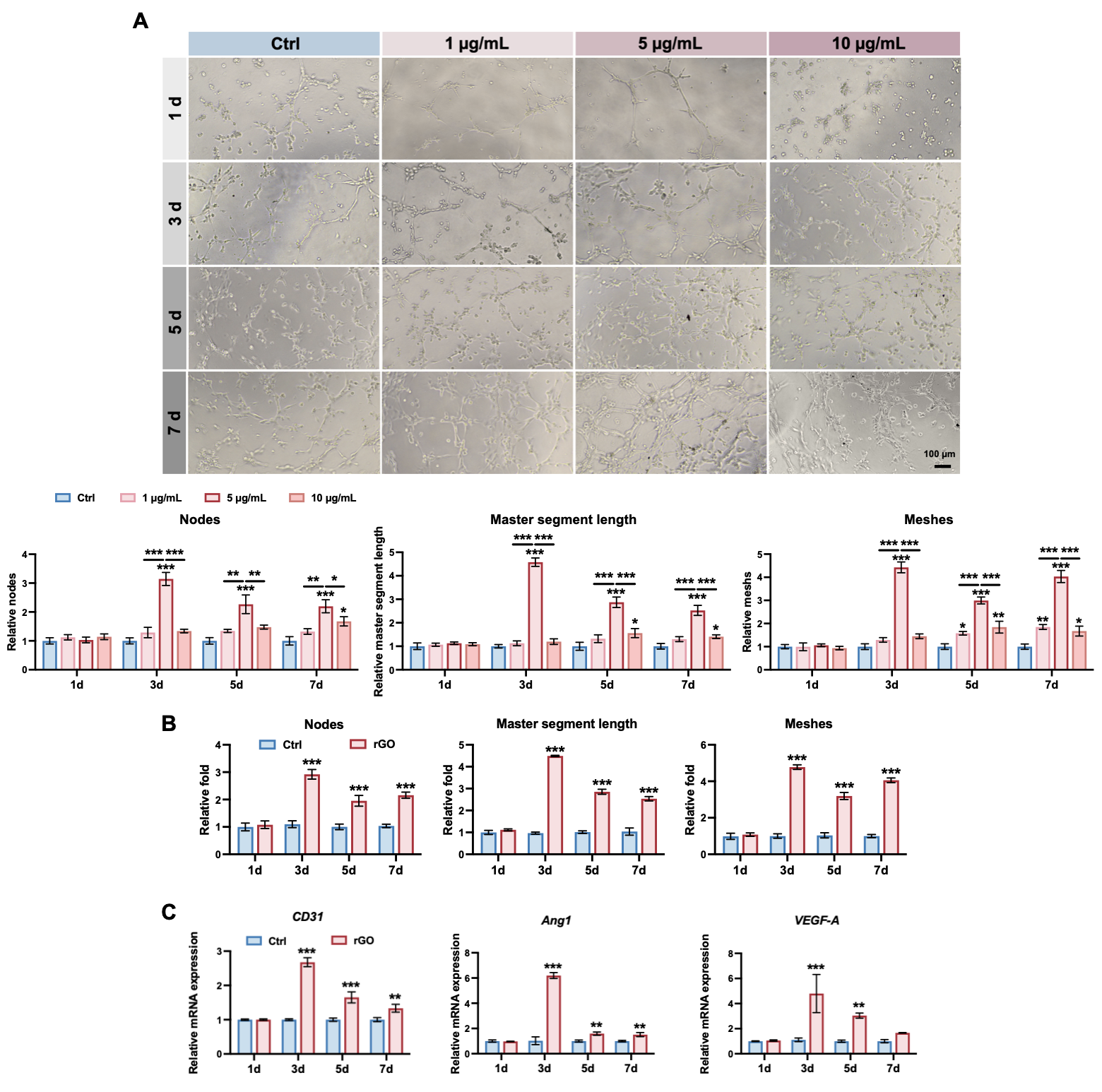


**Fig. S4** **rGO enhances the pro-angiogenic capacity of SHEDs**

(A) Representative images of tube formation by SHEDs treated with different concentrations of rGO for 1, 3, 5, or 7 d. (B) Quantification of total tube nodes, master segment length, and total meshes from the tube formation assay. (C) qRT-PCR analysis of angiogenesis-related gene expression (CD31, Ang1, VEGF-A) in SHEDs treated with 5 µg/mL rGO for the indicated durations. Data are presented as mean ± SD (n=3 per group). Statistical significance was determined using a one-way ANOVA with Tukey’s *post hoc* test for panels A and by a two-way ANOVA followed by Sidak’s multiple comparisons test for panel B, C. **P* < 0.05, ***P* < 0.01, and ****P* < 0.001.


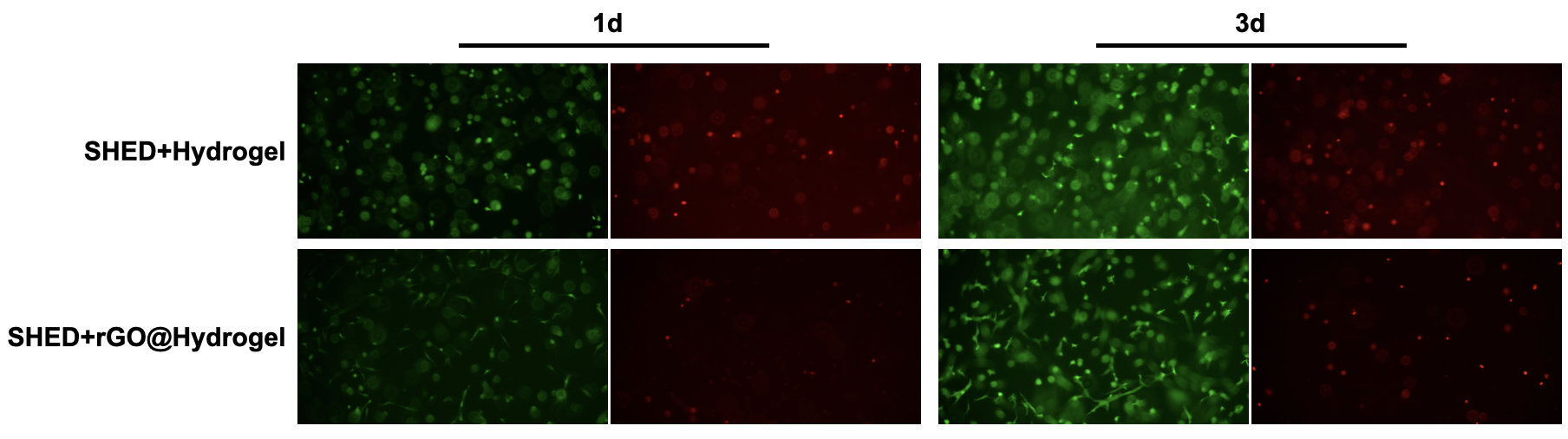


**Fig. S5** **SHEDs maintain high viability within the hydrogel matrix**

Representative fluorescence images of live/dead staining of SHEDs encapsulated in the hydrogel at 1 and 3 d. Live cells are stained green (Calcein-AM), and dead cells are stained red (PI).


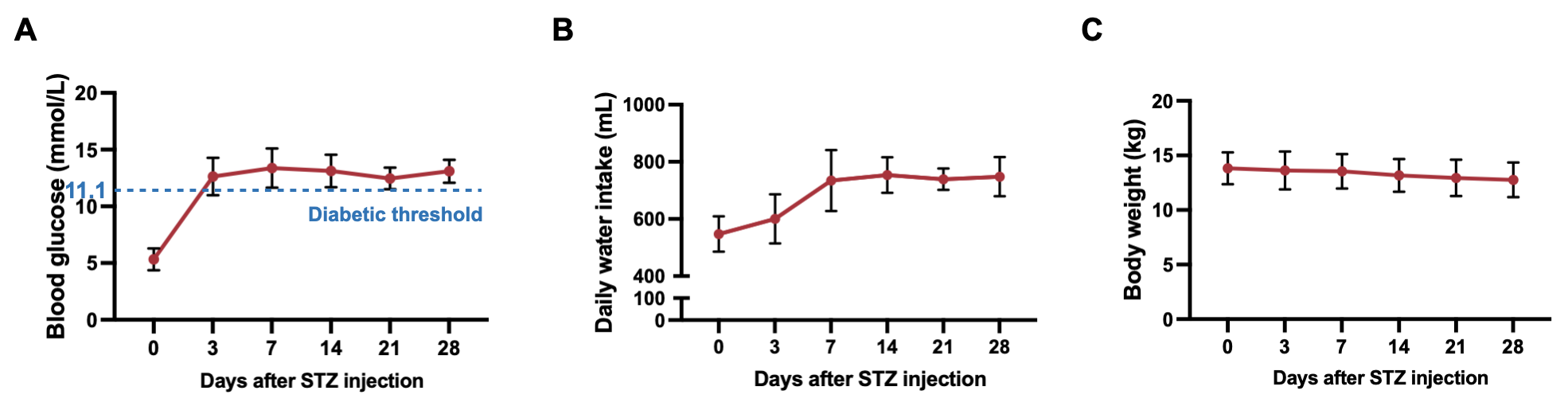


**Fig. S6** **Physiological characterization of the STZ-induced diabetic beagle dog model**

Following STZ injection in beagle dogs: (A) fasting blood glucose levels (diabetic threshold at 11.1 mmol/L shown by blue dashed line); (B) daily water intake; (C) body weight change over 28 days. Data are presented as mean ± SD (n=4 per group).


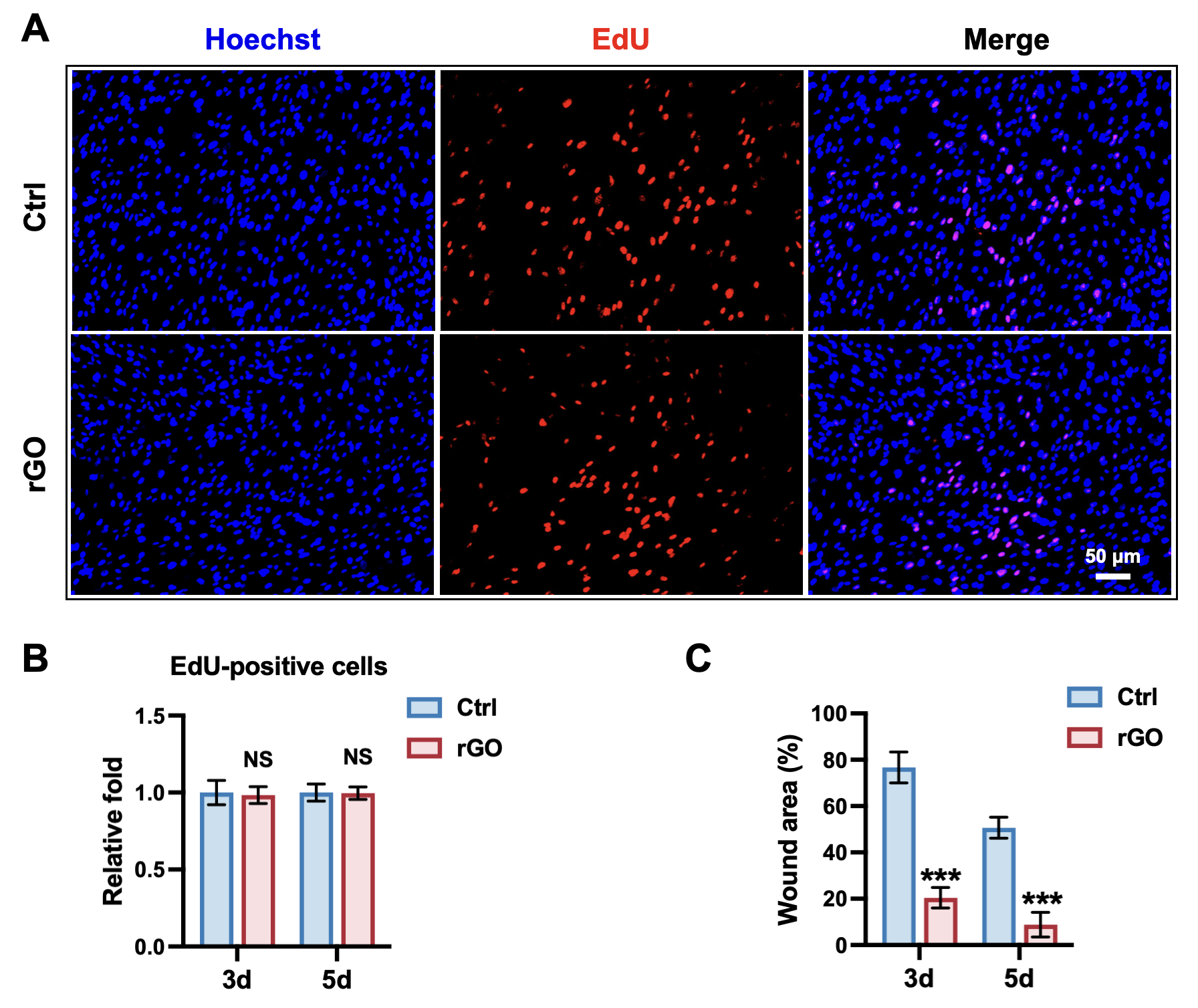


**Fig. S7** **rGO promotes SHED migration without affecting proliferation**

(A-B) Representative images and quantification of the EdU assay showing the proliferation rate of SHEDs after treatment with rGO for 3 and 5 d. (C) Quantification of the wound healing assay, showing the remaining wound area (%) in SHED monolayers treated with 5 µg/mL rGO at 3 and 5 d post-scratch. Data are presented as mean ± SD (n=3 per group). Statistical significance was determined by a two-way ANOVA followed by Sidak’s multiple comparisons test. NS: no significance. ****P* < 0.001.


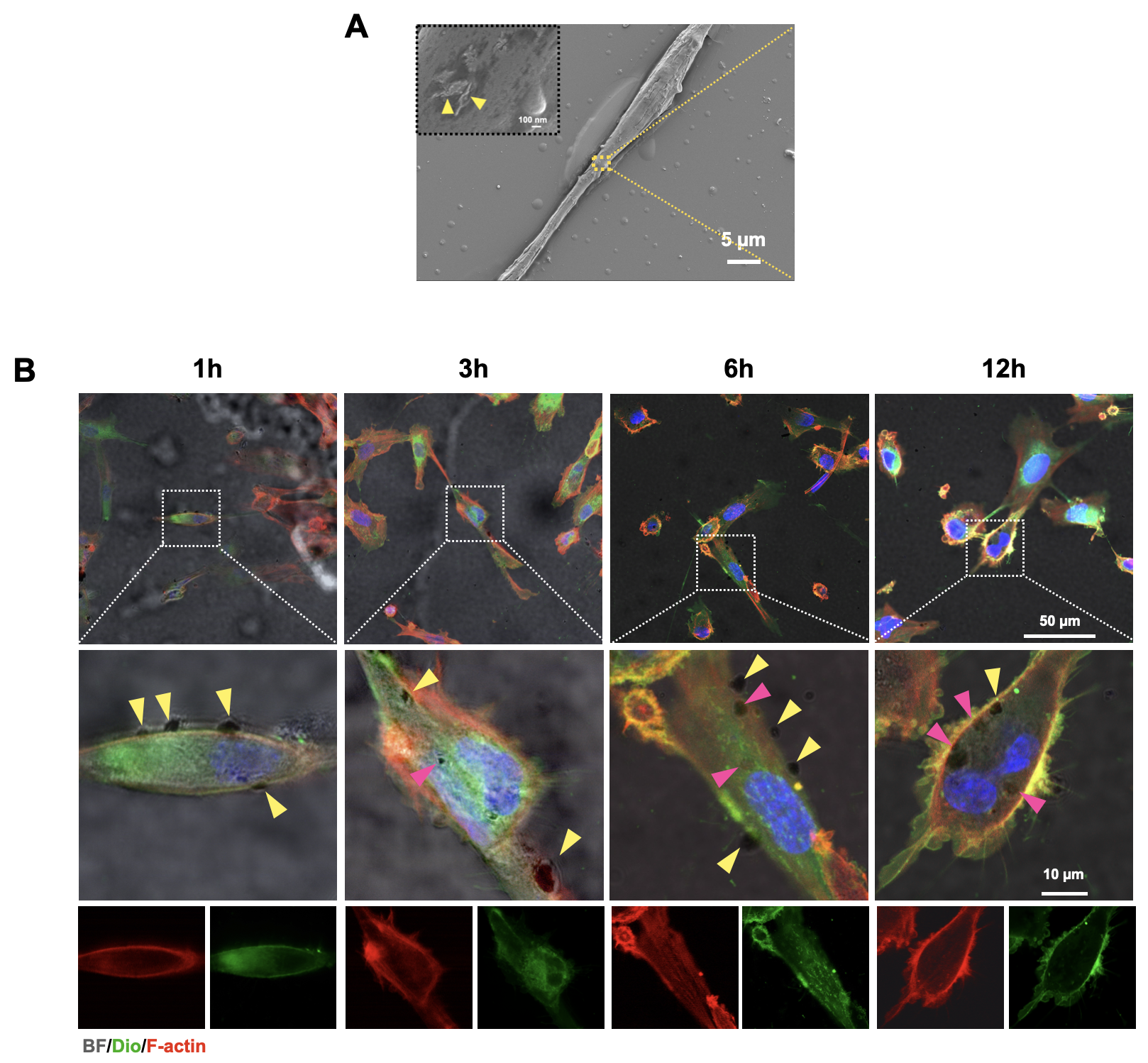


**Fig. S8** **rGO progresses from membrane attachment to cellular internalization**

(A) SEM imaging showing rGO sheets attached to or partially inserted into the SHED cell membrane (yellow arrows) after 0.5 h of incubation. Scale bar, 5 μm (overview) and 300 nm (magnified inset). (B) Fluorescence staining tracking the progressive internalization of rGO from membrane-associated (yellow arrows) to intracellular (pink arrows) localization over 12 h. Scale bar, 50 μm (overview) and 10 μm (magnified inset).


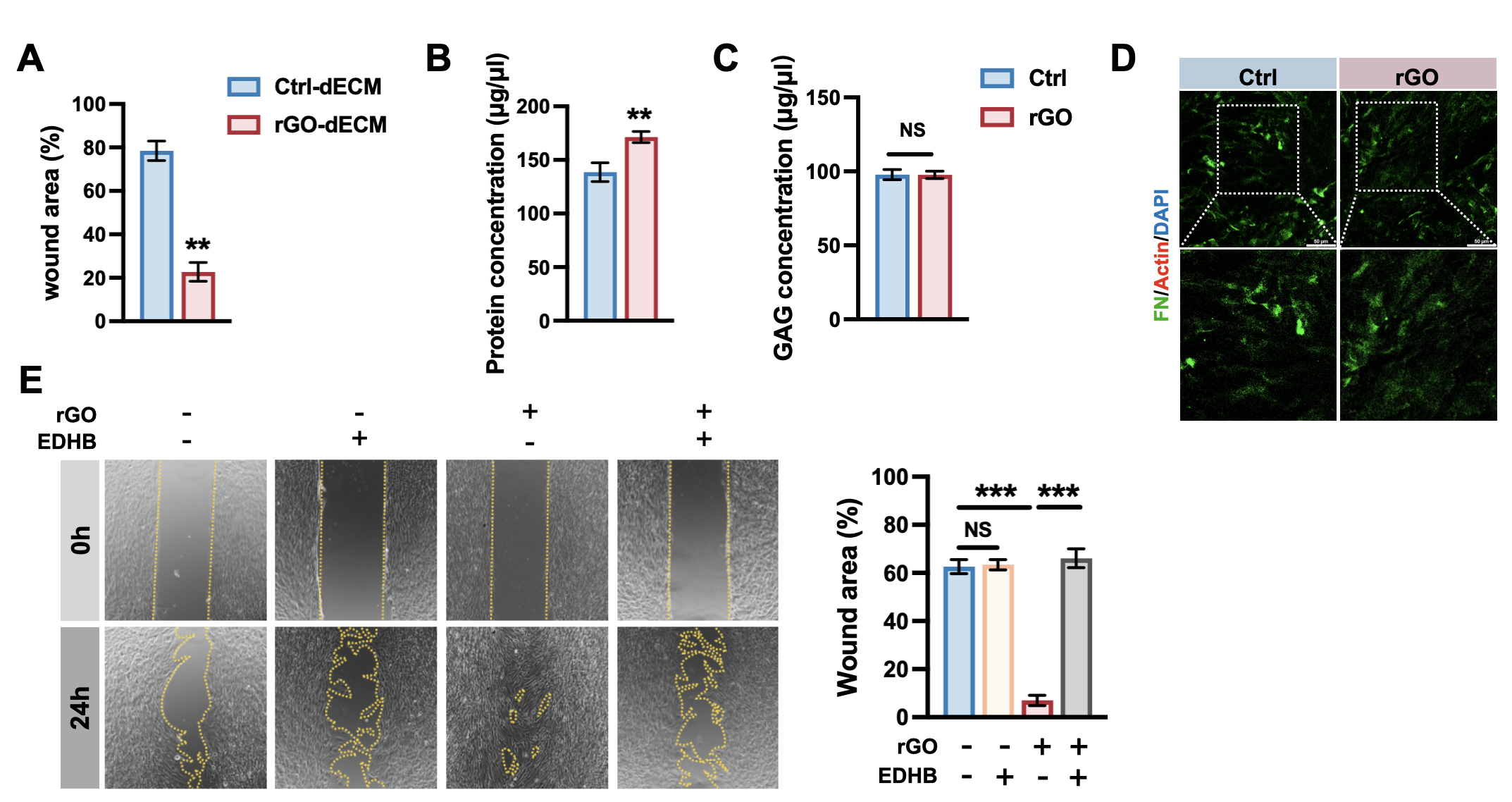


**Fig. S9** **rGO promotes ECM protein synthesis to enhance SHED migration**

(A) Quantification of wound healing assay of SHEDs cultured with dECM derived from rGO-treated cells. (B) BCA quantification demonstrating the total protein content in dECM treated with rGO. (C) DMMB assay quantifying GAG content in the dECM treated with rGO. (D) Immunofluorescence staining for fibronectin (FN) in the dECM. (E) Quantification of wound healing assay in SHEDs treated with or without the COL1A inhibitor EDHB. The wound area (%) was measured using ImageJ. Data are presented as mean ± SD (n=3 per group). Statistical significance was determined using two-tailed unpaired *t*-test for panel A, B, C and by a one-way ANOVA with Tukey’s *post hoc* test for panels E. NS: no significance. ***P* < 0.01, and ****P* < 0.001.


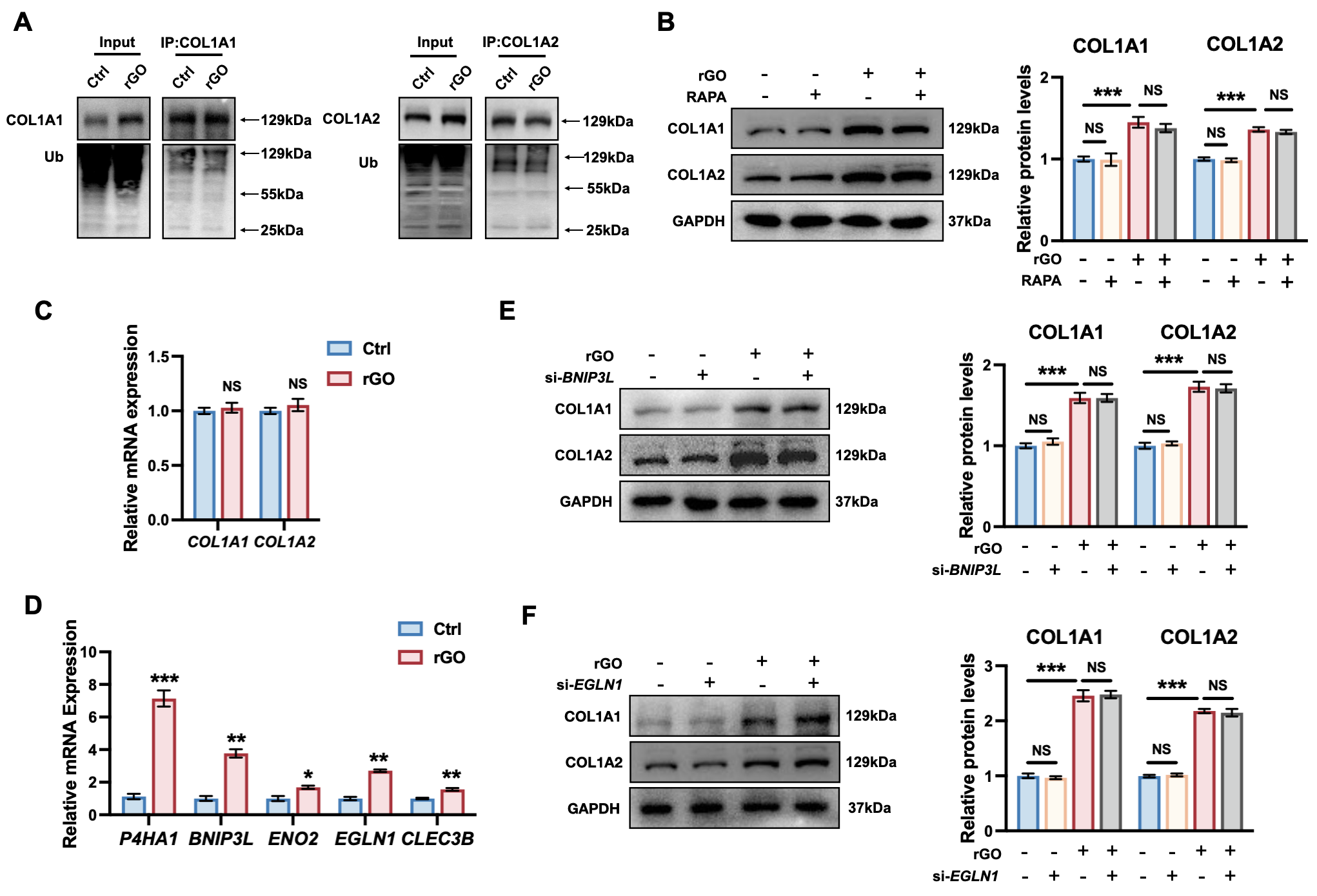


**Fig. S10** **rGO-induced increase in collagen I is regulated post-translationally via P4HA1**

(A) Western blot analysis of total protein ubiquitination in SHEDs treated with or without rGO, indicating that rGO does not alter global protein degradation via the ubiquitin-proteasome pathway. (B) Western blot analysis of COL1A1 and COL1A2 protein expression in SHEDs treated with the autophagy activator Rapamycin (RAPA) (C) RT-qPCR analysis of *COL1A1* and *COL1A*2 mRNA levels in SHEDs following rGO treatment. The data demonstrate that rGO does not upregulate collagen I at the transcriptional level. (D) RT-qPCR validation of the top five upregulated ER-associated genes (*P4HA1*, *BNIP3L*, *ENO2*, *EGLN1*, *CLEC3B*) identified from transcriptomic analysis in rGO-treated SHEDs. (E-F) Western blot analysis of COL1A1 and COL1A2 protein levels in SHEDs transfected with *BNIP3L* siRNA (si-*BNIP3L*) or *EGLN1* siRNA (si-*EGLN1*), followed by treatment with or without rGO. Data are presented as mean ± SD (n=3 per group). Statistical significance was determined using a one-way ANOVA with Tukey’s *post hoc* test for panels B, F, G and by two-tailed unpaired *t*-test for panel C, D. NS: no significance. **P* < 0.05, ***P* < 0.01, and ****P* < 0.001.


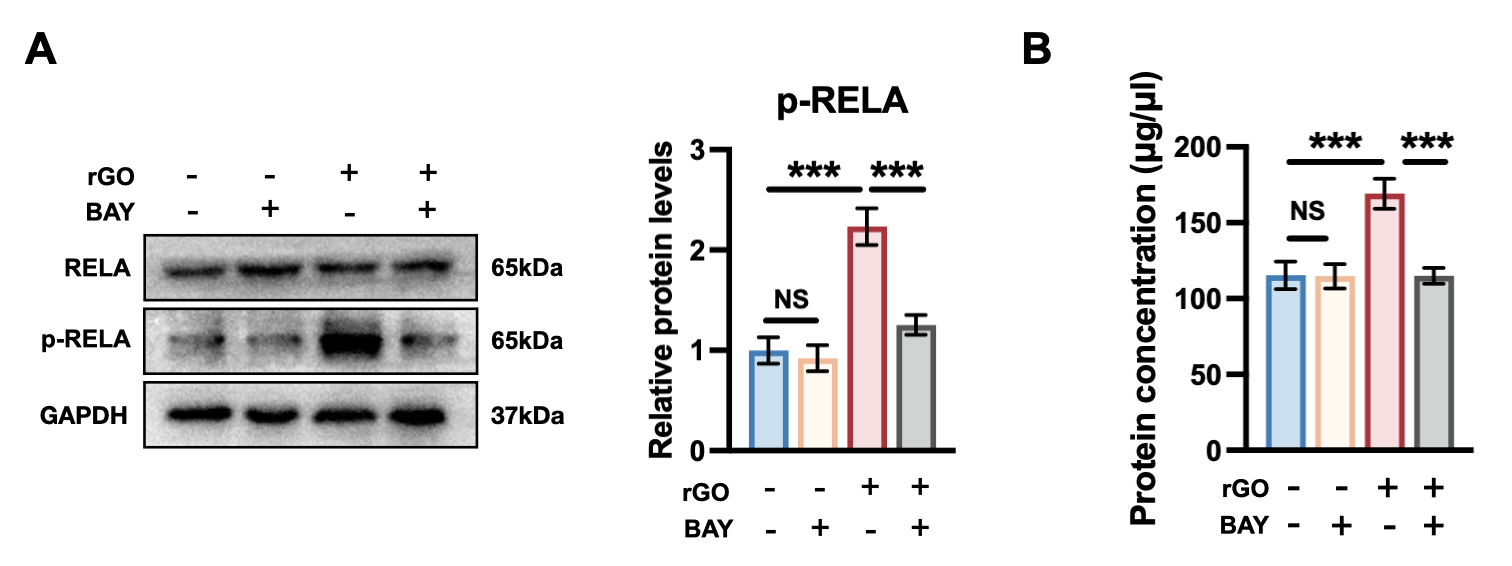


**Fig. S11 RELA is the upstream transcription factor of P4HA1**

SHEDs were treated with rGO with or without the RELA inhibitor BAY. (A) Western blot showing the levels of RELA and p-RELA. Changes in phosphorylated protein levels were quantified after normalization to the corresponding total protein. (B) BCA quantification demonstrating the total protein content in dECM produced by rGO-treated cells. Data are presented as mean ± SD (n=3 per group). Statistical significance was determined using a one-way ANOVA with Tukey’s *post hoc* test. NS: no significance. ****P* < 0.001.


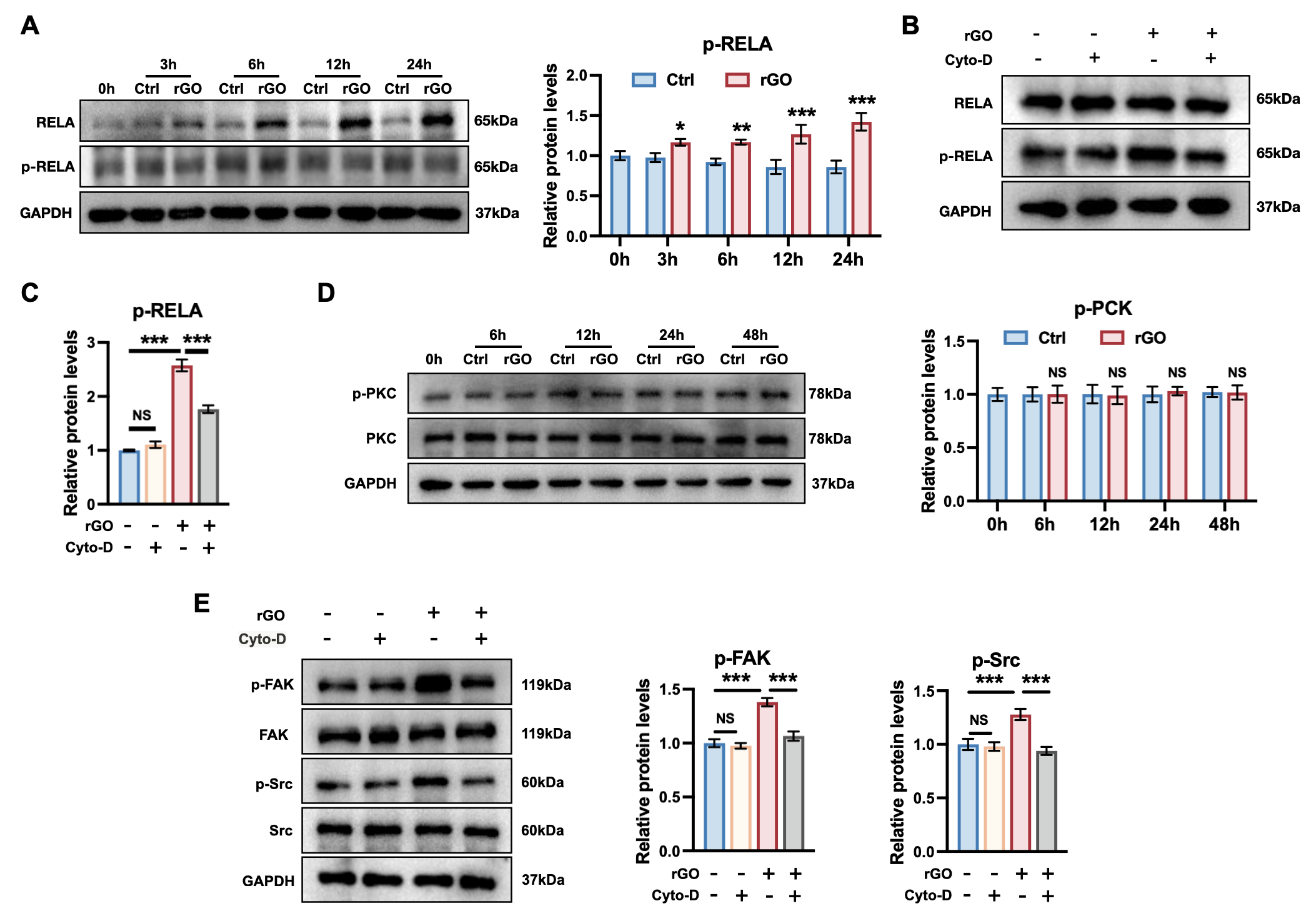


**Fig. S12** **rGO-induced RELA phosphorylation requires actin dynamics and is mediated by FAK/Src signaling**

(A) Time-course Western blot of p-RELA and RELA levels after rGO treatment at 3, 6, 12 and 24 h. (B-C) Western blot analysis and quantification of p-RELA/RELA ratio in SHEDs treated with rGO with or without the actin polymerization inhibitor Cytochalasin D (Cyto-D). (D) Western blot showing the levels of PKC and p-PKC. (E) Western blot analysis demonstrating that the phosphorylation of FAK and Src was abrogated by Cyto-D. Data are presented as mean ± SD (n=3 per group). Statistical significance was determined using two-tailed unpaired *t*-test for panel A, D and by a one-way ANOVA with Tukey’s *post hoc* test for panels C, E. NS: no significance. **P* < 0.05, ***P* < 0.01, ****P* < 0.001.


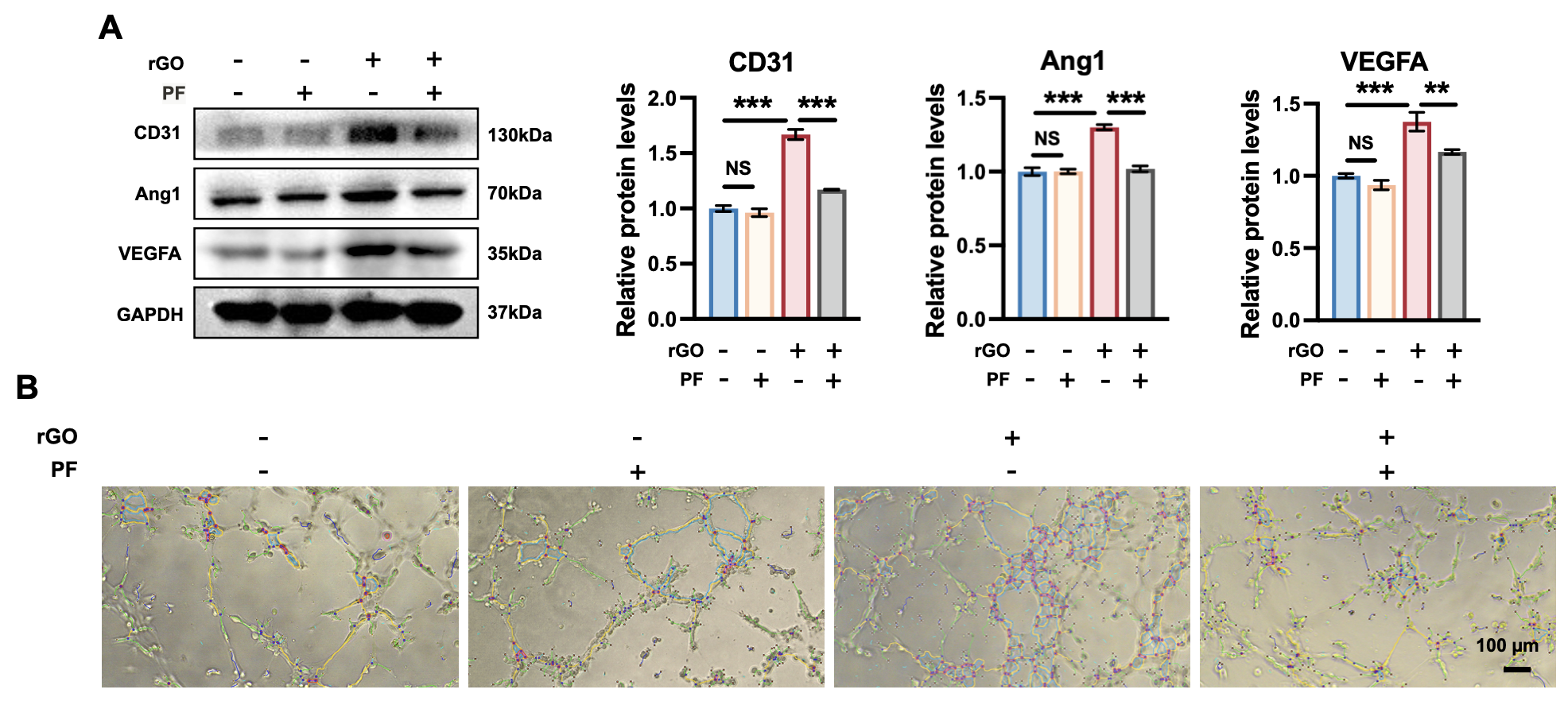


**Fig. S13** **FAK signaling is essential for rGO-induced pro-angiogenic functions**

SHEDs were treated with rGO in the presence or absence of the FAK inhibitor PF-573228 (PF). (A) Western blot analysis of angiogenesis-related protein expression. (B) Representative images of the tube formation assay. Data are presented as mean ± SD (n=3 per group). Statistical significance was determined using a one-way ANOVA with Tukey’s *post hoc* test. NS: no significance. ***P* < 0.01 and ****P* < 0.001.


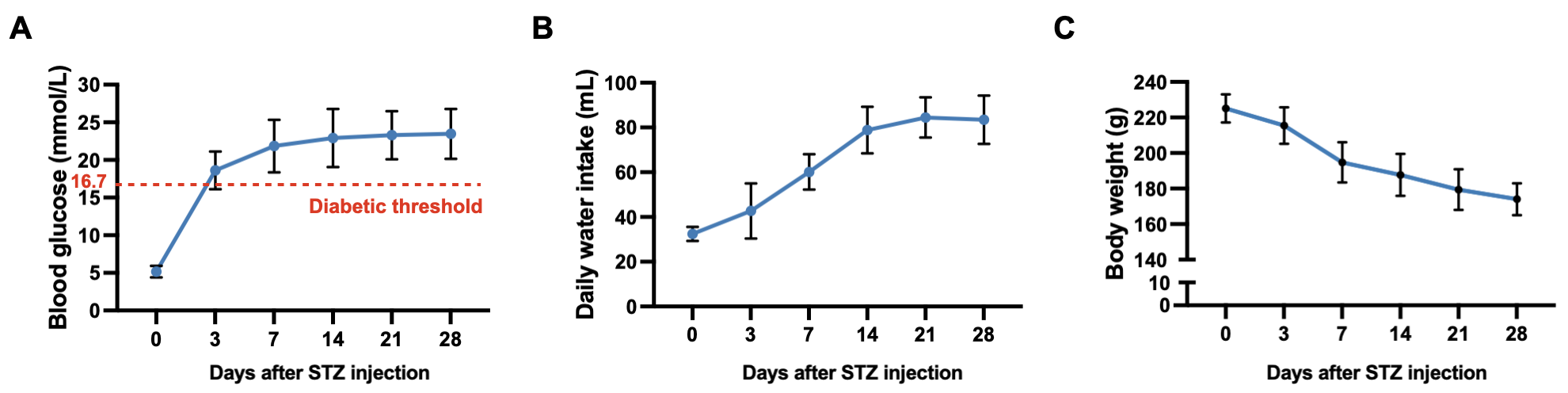


**Fig. S14** **Physiological characterization of the STZ-induced rat model**

Following STZ injection in SD rats: (A) fasting blood glucose levels (diabetic threshold at 16.7 mmol/L shown by red dashed line); (B) daily water intake; (C) body weight change over 28 days. Data are presented as mean ± SD (n=45 per group).


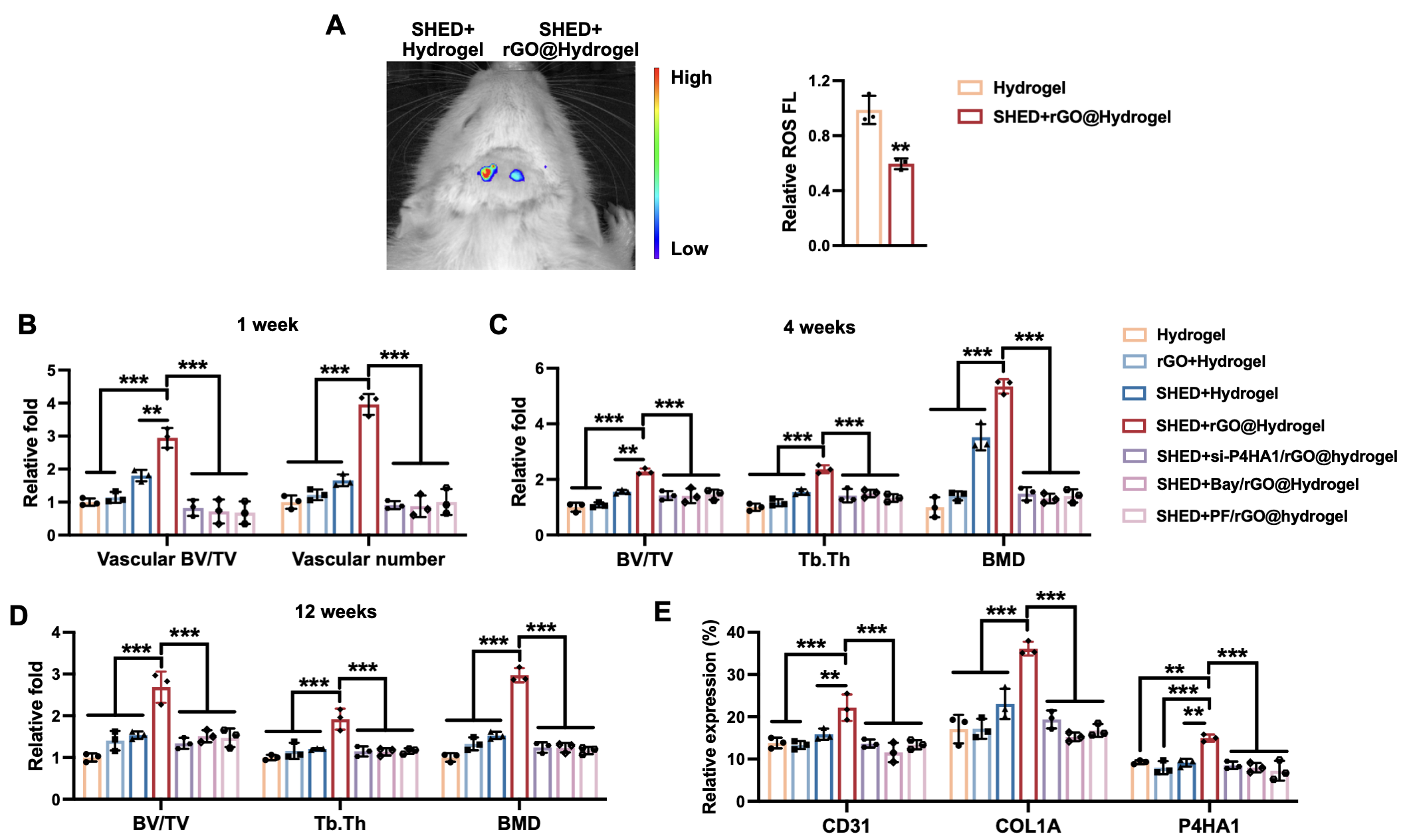


**Fig. S15** **Quantitative analysis of *in vivo* angiogenesis and bone regeneration in a diabetic rat calvarial defect model**

(A) Representative *in vivo* fluorescence images of SD rat calvarial defects at 4 weeks post-surgery, visualing ROS levels in the SHED+Hydrogel and SHED+rGO@Hydrogel groups with the ROS Brite^TM^ 700 probe. (B) Quantitative analysis of angiogenesis and bone regeneration in diabetic rat calvarial defects following implantation of prevascularized scaffolds at 1, 4 and 12 weeks, as assessed by Micro-CT. (C) The bar chart illustrating quantitative immunohistochemical analysis of CD31, COL1A and P4HA1 expression in hydrogels and surrounding tissues at 4 weeks post-implantation. Data are presented as mean ± SD (n=3 per group). Statistical significance was determined using two-tailed unpaired *t*-test for panel A and by a one-way ANOVA with Tukey’s *post hoc* test for panels B-E. ***P* < 0.01 and ****P* < 0.001.


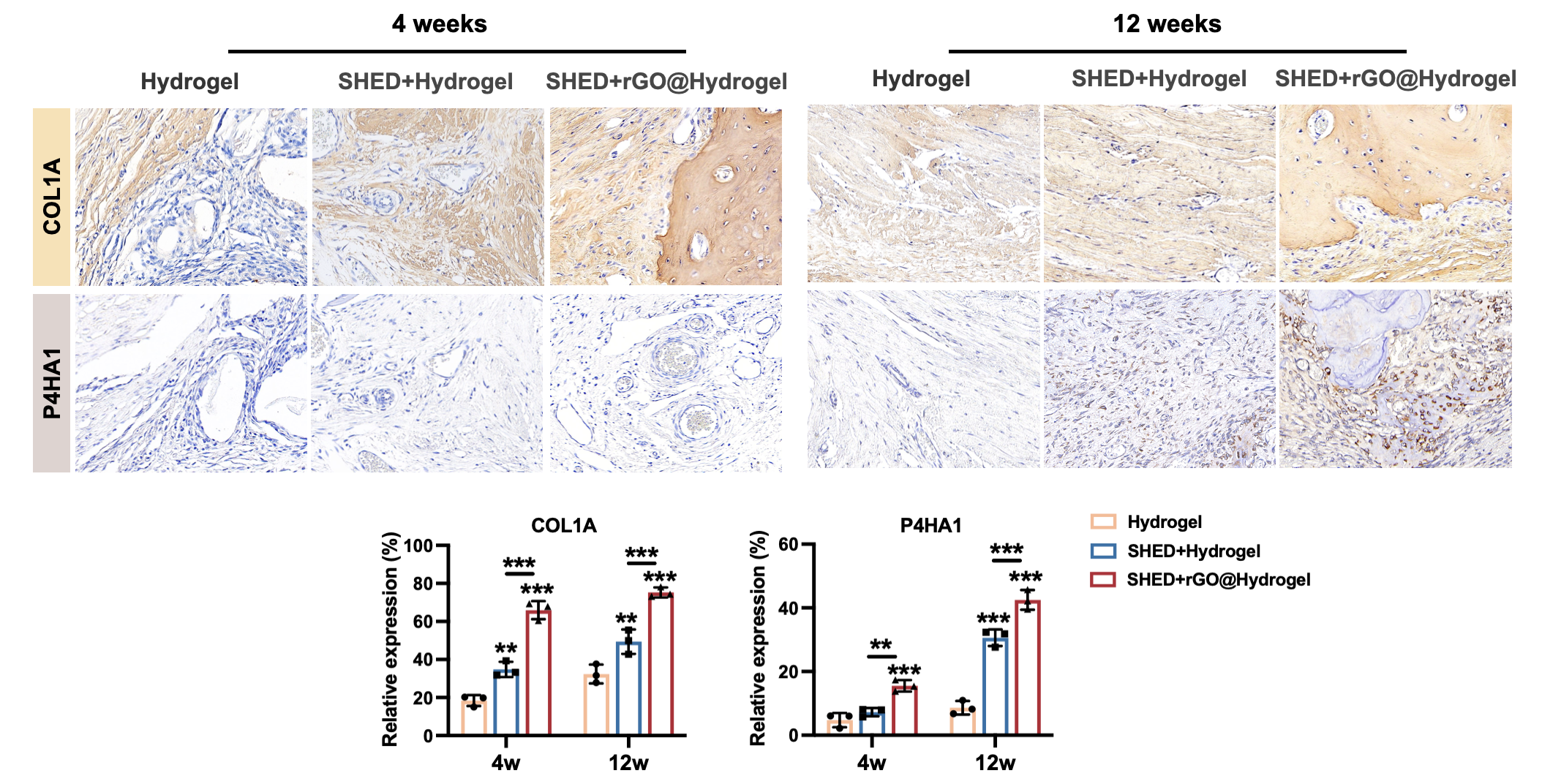


**Fig. S16** **The** **prevascularized rGO@Hydrogel promotes ECM remodeling via P4HA1/ COL1A1 in a diabetic large-animal model**

Immunohistochemical staining of P4HA1 and COL1A in hydrogels and surrounding tissues of diabetic beagle mandibular bone defects at 4 and 12 weeks after implantation of different hydrogel groups. Data are presented as mean ± SD (n=3 per group). Statistical significance was determined using a two-way ANOVA followed by Sidak’s multiple comparisons test. ***P* < 0.01, and ****P* < 0.001.

**Tables**

**Tab. S1** **Antibodies used in this study**

| **Antibody** | **Host** | **Catalog** | **Dilution ratio** | **Vendor** |
| --- | --- | --- | --- | --- |
| Anti-vWF | Rabbit | 27186-1-AP | 1:5000 for wb | Proteintech |
| Anti-KDR | Rabbit | 26415-1-AP | 1:5000 for wb | Proteintech |
| Anti-CD31 | Rabbit | 11265-1-AP | 1:1000 for wb  1:500 for IF  1:500 for IHC | Proteintech |
| Anti-Ang-1 | Rabbit | 68618-1-AP | 1:1000 for wb | Proteintech |
| Anti-VEGFA | Rabbit | 19003-1-AP | 1:1000 for wb | Proteintech |
| Anti-Collagen I | Rabbit | AF7001 | 1:500 for IHC  1:1000 for wb  1:500 for IF | Affinity |
| Anti- Collagen I alpha 2 | Rabbit | DF3549 | 1:500 for IHC  1:1500 for wb  1:500 for IF | Affinity |
| Anti-Collagen III | Rabbit | AF5457 | 1:1000 for wb | Affinity |
| Anti-Collagen IV | Rabbit | AF0510 | 1:500 for IHC  1:500 for wb | Affinity |
| Anti-Fibronectin | Rabbit | ET1702-25 | 1:1500 for wb | HUABIO |
| Anti-P4HA1 | Rabbit | 12658-1-AP | 1:2000 for IHC  1:5000 for wb | Proteintech |
| Anti-BNIP3L | Rabbit | A6283 | 1:1000 for wb | ABclonal |
| Anti-ENO2 | Rabbit | A12341 | 1:1000 for wb | ABclonal |
| Anti-EGLN1 | Rabbit | A10342 | 1:1000 for wb | ABclonal |
| Anti-CLEC3B | Rabbit | A4387 | 1:1500 for wb | ABclonal |
| Anti-NF-κB p65 | Rabbit | 10745-1-AP | 1:1000 for wb  1:500 for IF | Proteintech |
| Anti-STAT1 | Rabbit | A12075 | 1:1000 for wb  1:100 for IF | ABclonal |
| Anti-lamin B1 | Rabbit | 12987-1-AP | 1:1000 for wb | Proteintech |
| Anti-Phospho-NF-κB p65 (Ser536) | Rabbit | 3033T | 1:1000 for wb | Cell Signaling Technology |
| Anti-NF-κB p65  (acetyl K310) | Rabbit | Ab19870 | 1:1500 for wb | Abcam |
| Anti-actin | Mouse | M1210-2 | 1:30000 for wb | HUABIO |
| Anti-FAK | Rabbit | ET1602-25 | 1:5000 for wb | HUABIO |
| Anti- Phospho-FAK | Rabbit | ET1610-34 | 1:1000 for wb | HUABIO |
| Anti-Src | Rabbit | AF6161 | 1:1000 for wb | Affinity |
| Anti- Phospho-Src (Tyr419) | Rabbit | AF3162 | 1:1000 for wb | Affinity |
| Anti-PKC | Rabbit | ET1701-85 | 1:3000 for wb | HUABIO |
| Anti- Phospho-PKC | Rabbit | HA722458 | 1:1000 for wb | HUABIO |
| Anti-Gapdh | Mouse | MAB45855 | 1:1000 for wb | Biosharp |
| Goat anti-Rabbit IgG (HRP Conjugate) | Goat | HA1001 | 1:50000 for wb | HUABIO |
| Goat anti-Mouse IgG (HRP Conjugate) | Goat | HA1006 | 1:50000 for wb | HUABIO |
| Goat anti- Rabbit IgG (594 Conjugate) | Goat | AS039 | 1:100 for IF | ABclonal |

**Tab. S2 Primers used in this study**

| **Name** | **Sequence** |
| --- | --- |
| *CD31*-F | GTAAGGTGGTGGAGTCTGGAGAG |
| *CD31-*R | TGGGTGGCATTTGAGGTCATTTG |
| *Ang-1*-F | CGCTGCCATTCTGACTCACATAG |
| *Ang-1*-R | GTACTCTCACGACAGTTGCCATC |
| *VEGFA*-F | TGCTGCTCTACCTCCACCATG |
| *VEGFA*-R | ATGATTCTGCCCTCCTCCTTCTG |
| *P4HA1*-F | AACGGCTGAGGACTGCTTTGA |
| *P4HA1*-R | ATCTCGCCTTCATCCAGTTGC |
| *Col1A1*-F | GATTCCCTGGACCTAAAGGTGC |
| *Col1A1*-R | AGCCTCTCCATCTTTGCCAGCA |
| *Col1A2*-F | TGGCAAAGAAGGCGGCAAAG |
| *Col1A2*-R | GAGGAGCACCAGCAGGACCAT |
| *BNIP3L-F*  *BNIP3L-R*  *ENO2-F*  *ENO2-R*  *EGLN1-F*  *EGLN1-R*  *CLEC3B-F*  *CLEC3B-R*  *S18-*F  *S18-R* | AGCAGGGACCATAGCTCTCA  CAGTCTGATACCCAGTCCGC  TGCCCCCAATATCCTGGAGA  ATCGGGAAGGATCAGTGGGA  CTCGTCCAAGGACATCCGAG  TCATGAGCAGCCCAATGGTT  CCCAGACGAAGACCTTCCAC  GGTACTCATACAGGGCGTCG  TGCCCTCTGCTCCTCTCTTC  CCAGCTTCTCCATGGTGATG |
